# Functional hyperemia drives fluid exchange in the paravascular space

**DOI:** 10.1186/s12987-020-00214-3

**Published:** 2020-08-20

**Authors:** Ravi Teja Kedarasetti, Kevin L. Turner, Christina Echagarruga, Bruce J. Gluckman, Patrick J. Drew, Francesco Costanzo

**Affiliations:** 1 Center for Neural Engineering, The Pennsylvania State University, University Park, PA USA; 2 Department of Engineering Science and Mechanics, The Pennsylvania State University, University Park, PA USA; 3 Department of Neurosurgery, The Pennsylvania State University, University Park, PA USA; 4 Department of Biomedical Engineering, The Pennsylvania State University, University Park, PA USA; 5 Department of Mathematics, The Pennsylvania State University, University Park, PA USA

## Abstract

The brain lacks a conventional lymphatic system to remove metabolic waste. It has been proposed that directional fluid movement through the arteriolar paravascular space (PVS) promotes metabolite clearance. We performed simulations to examine if arteriolar pulsations and dilations can drive directional CSF flow in the PVS and found that arteriolar wall movements do not drive directional CSF flow. We propose an alternative method of metabolite clearance from the PVS, namely fluid exchange between the PVS and the subarachnoid space (SAS). In simulations with compliant brain tissue, arteriolar pulsations did not drive appreciable fluid exchange between the PVS and the SAS. However, when the arteriole dilated, as seen during functional hyperemia, there was a marked exchange of fluid. Simulations suggest that functional hyperemia may serve to increase metabolite clearance from the PVS. We measured blood vessels and brain tissue displacement simultaneously in awake, head-fixed mice using two-photon microscopy. These measurements showed that brain deforms in response to pressure changes in PVS, consistent with our simulations. Our results show that the deformability of the brain tissue needs to be accounted for when studying fluid flow and metabolite transport.

## Introduction

The brain is surrounded by cerebrospinal fluid (CSF), and the movement of CSF can clear metabolites from the central nervous system (CNS) [1–7]. The nature of CSF movement into the brain tissue (where it might exchange solutes and fluid with interstitial fluid, ISF) has been a source of controversy [8–15]. Recent work [16, 17] has suggested directional CSF movement along the paravascular space (PVS) around arterioles. The PVS is a fluid-filled region between the arteriolar smooth muscle and astrocyte endfeet, and is believed to be connected to the sub-arachnoid space (SAS). The bulk movement of CSF in the SAS is thought to be driven by heart beat-driven pulsations of arterioles, which pump CSF through the PVS of pial arteries (“peristaltic pumping”) [16, 18, 19], the production of CSF by the choroid plexus [20], or the volume changes of the brain from heartbeat [21] and respiration [22]. Because the PVS extends into the brain tissue along penetrating blood vessels, the conservation of fluid mass (continuity equation of fluid dynamics) dictates that directional flow in the PVS implies bulk flow of fluid in the parenchyma [16, 23]. However, several studies have given a conflicting view, claiming that there is no bulk fluid movement in the parenchyma (and in the PVS surrounding penetrating arterioles [13]), and that transport in the brain parenchyma is due to diffusion [10, 12, 14, 24, 25]. While the existence bulk fluid flow in brain parenchyma is controversial, there is clear agreement that there is bulk flow of CSF through the SAS, and that CSF exits from the cranial space through pathways in the dural sinuses, the base of the skull, or the cribriform plate [1–7, 26, 27].

An important approach for understanding fluid movement in the brain and the PVS is simulation of fluid dynamics. Calculations based on fluid mechanics [13, 19, 28] do not agree on the magnitude and direction of the proposed “peristaltic pumping” mechanism. Moreover, the previously published models have treated the brain tissue as a non-compliant solid for simplicity [11, 13, 14, 19]. In reality, brain tissue is very compliant [29–32] (‘soft’), with a shear modulus in the range of 1–8 kPa. Given the compliant nature of the brain, even relatively small pressure changes will cause deformations of the tissue and would consequently produce fluid movements very different from those that would occur if the brain were non-compliant. While the deformability of the brain tissue has been shown to affect several aspects of the CSF flow [21, 33–37], to the best of our knowledge, ours is the first model to investigate the effect of brain deformability on the flow in the PVS.

The study is organized into three parts. In the first part, we investigated the possibility of directional fluid movement in the PVS driven by the movement of arteriolar walls. We found that neither arteriolar pulsations, nor functional hyperemia could drive appreciable directional flow of CSF through the PVS. Next, we examined the possibility of metabolite clearance from the PVS through exchange of fluid between the PVS and SAS. Our simulations showed that functional hyperemia, but not arteriolar pulsations, can drive large fluid exchange between the PVS and the SAS. This fluid exchange could help remove metabolites from the CNS. Our simulations predicted that the difference in fluid exchange driven by pulsations and hyperemia are partly because of the deformation of brain tissue in response to the pressure changes in the PVS. We verified this prediction by using in vivo two-photon microscopy to measure the deformation of the brain tissue and arteriolar diameter during functional hyperemia. Our measurements revealed that the brain tissue can in-fact deform from pressure changes in the PVS, as suggested by our model. These results suggest that in addition to its involvement in other processes, functional hyperemia can aid metabolite clearance in the brain through the exchange of CSF between the SAS and PVS.

### Modeling choices

We first explain our modeling choices and parameters before diving into the results of our simulations. We are interested in understanding how the motions of the arteriolar walls drive fluid exchange between the PVS and SAS. We performed fluid mechanics simulations of the CSF in the PVS surrounding penetrating arterioles in adult mice. There is great deal of ambiguity regarding several key parameters governing the fluid flow of the PVS, namely the permeability of the PVS, channel width of the PVS, and the flow resistance of the surrounding spaces (the brain parenchyma and the SAS). These ambiguities pertaining to flow in the PVS within the subarachnoid space [23] and the parenchyma [38] are discussed in detail in recent reviews on the subject. Keeping these ambiguities in mind, we performed simulations using a wide range of parameters (see Table 1) to ensure that our results are robust.

**Table 1:** Parameters used in simulations

Parameter name	Symbol	Default	Range	Unit	Source
Arteriolar radius	R_1_	12	5 to 20	µm	[49– 51]
PVS length	L_a_	250	250 to 500	µm	[50, 145]
PVS width	wd	3	2–10	µm	[16, 42, 43]
CSF viscosity	µ_f_	0.001	–	Pa.s	[47, 48]
CSF density	ρ_f_	1000	-	kg/m^3^	[47, 48]
PVS porosity	ζ	0.8	0.5–0.9	–	[16, 17, 146]
PVS permeability	k_s_	2 × 10^−14^	7 × 10^−13^ to 2 × 10^−15^	m^2^	[12, 14, 45, 46]
Brain section radius	R_3_	150	100–200	µm	[145, 147]
Brain shear modulus	µ_s_	4	1–8	kPa	[29–32, 63–66]
Brain tissue density	ρ_1_	1000	–	kg/m^3^	[148]
Pulsation amplitude (% arteriolar radius)	b_1_	1	0.5–2	–	[16]
Pulsation frequency	f	10	7–14	Hz	[72]
Pulse wave speed	c	1	0.5–10	m/s	[69–71]
Pulse wave wavelength	λ	0.1	0.03–1.43	m	c/f
Diffusion coefficient	D	1.4 × 10^−6^	–	cm^2^/s	[134, 135]

We posit that fluid movement in the PVS is governed by the Darcy-Brinkman [39] equations (one for the momentum balance and the other for volume conservation), which is used to simulate flow through highly porous regions [40]. This choice is based on the experimental data available from recent studies that used intra-cisternal infusions to study the flow of CSF. These studies have shown unobstructed movement of 1 µm particles in the PVS surrounding arterioles on the surface of the brain [16, 17]. While these relatively large particles do not enter the PVS surrounding penetrating arterioles, dye-conjugated dextrans (3–500 kDa) with a hydrodynamic radius of 1–15 nm [41] have been shown to enter the PVS around the penetrating arterioles [42–44]. Based on these results, we modeled the PVS surrounding penetrating arterioles as a porous medium with higher porosity and fluid permeability than the brain tissue. The porosity (fraction of fluid volume to the total volume) of the PVS was modelled to be between 0.5 and 0.9. The fluid permeability of the PVS is taken from a range of possible values. The minimum value of PVS permeability we used was 2 × 10^−15^ m^2^, the measured permeability of the brain tissue [45, 46]. We also performed simulations with infinite permeability, where the Darcy-Brinkman equations recover the standard Navier–Stokes equations that govern flow in an open channel. The default value of permeability was taken to be 2 × 10^−14^ m^2^, where the PVS is 10 times more permeable than the brain parenchyma. This was chosen because dye injected through the cisterna magna enters the PVS nearly 10 times faster than it enters the parenchyma (< 5 min vs. ~ 30 min) [43], presumably under a single source of difference in fluid pressure. The viscosity (0.001 Pa*s) and density (1000 kg/m^3^) of CSF were taken from experimentally determined values [47, 48].

The PVS is assumed to be 150–300 µm long and 2–10 µm wide for an arteriolar radius of 5–20 µm [49–51]. The length of 150–300 µm is in the range of bifurcation free length of penetrating arterioles in the mouse parenchyma. This is consistent with the length of the PVS used in previous studies that used an axisymmetric model of the PVS surrounding penetrating arterioles [13]. While the width of the PVS surrounding large pial arteries is in the range of 20–40 µm [16, 17], the PVS around penetrating arterioles appears to be much smaller (this is clearly evident in Fig. 6c of Schain et al. [42]). The width of this section of the PVS is not explicitly mentioned in the literature. However, a width of 2–10 µm can be calculated from the imaging data available from experimental studies [42, 43].

**Fig. 1:**
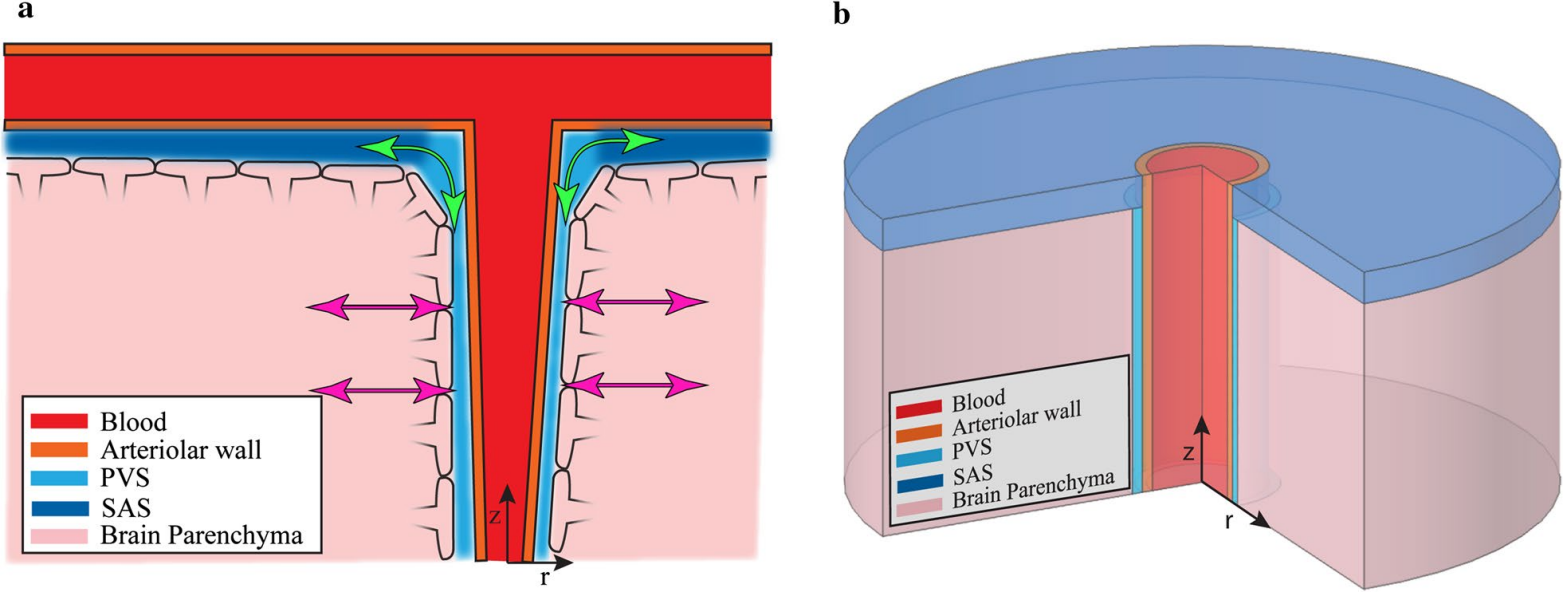
Schematic of the anatomical structure of a penetrating arteriole and surrounding tissue. **a** Depiction the fluid filled PVS between the arteriolar wall and the brain parenchyma. adapted from Abbot et al. [10]. The glia limitans covers the surface of the brain tissue and forms the brain-PVS interface. The subarachnoid space (SAS) and paravascular spaces (PVS) are interconnected fluid-filled compartments. The low resistance pathway for fluid flow to and from the PVS (along the SAS) is shown in green, while the high resistance pathway (through the brain parenchyma) is shown in magenta. **b** Geometry of the computational model of a penetrating arteriole and the brain and fluid around it. The model is cylindrically symmetric around the penetrating arteriole, allowing us to use axisymmetric simulations (see Additional file 1: Appendix for full mathematical detail)

In the cerebral cortex of mice, the fluid leaving the PVS around penetrating arterioles has to enter the SAS or the PVS around pial arteries on the pial side (green arrows in Fig. 1a) or the brain parenchyma or the para-capillary and para-venous spaces on the other side (magenta arrows in Fig. 1a). To avoid confusion, we refer to the first set of fluid chambers as the SAS and the second set as the parenchyma. Due to the relatively large PVS surrounding the pial vessels [16, 17], the SAS region has a relatively low flow resistance compared to the PVS. Therefore, in our models the pial opening of the PVS is connected to a flow resistance with a resistance value 1/100th (or 1/10th, see results) of the flow resistance of the PVS. The parenchyma is assumed to have a higher flow resistance, 10 times that of the PVS. There is evidence suggesting the anatomy, and therefore the flow path of CSF, is more complicated than what we modelled here. Potter et al. [52, 53] showed that the PVS might be very small or non-existent in the healthy brain. Albargothy et al. [54] showed that CSF in the para-arterial space mostly likely flows out through the periarterial basement membrane and not out of the paravenous space. There is also evidence showing that the PVS and the SAS are not contiguous fluid filled compartments but are connected through the stomata or pores in the leptomeningeal cell layer surrounding arterioles [55, 56]. Even though our assumptions do not exactly match these findings, the path of least resistance for the flow of CSF seems to be through the SAS and around penetrating arterioles [52–56], which was captured in our models.

We did not explicitly model fluid flow into the brain parenchyma, either through possible gaps in the glia limitans surrounding arterioles [57] or through the aquaporin channels in the astrocytic endfeet [43, 58, 59], because there is no agreement on the existence of bulk flow in the brain parenchyma [8, 10–12, 14, 16, 59]. Models that have simulated the flow through astrocytic endfeet and the brain extracellular space concluded that transport through these pathways is dominated by diffusion and not bulk flow [11, 14]. The flow through all these pathways is lumped together in a single effective parenchymal flow resistance. This is a limitation of our model, and our calculations of net flow into the brain need to be interpreted with this limitation in mind.

In models where we simulate the brain tissue as a deformable solid, the brain tissue was modelled as a compressible, Saint–Venant-Kirchhoff solid. A Poisson’s ratio of 0.45 was chosen, to match the known mechanical response of brain tissue under compression [60]. We also performed these simulations with an incompressible Neo-Hookean elastic model for the brain tissue. These Saint–Venant-Kirchhoff and Neo-Hookean models are chosen to minimize the number of model parameters. These models have been shown to accurately estimate brain tissue deformation during craniotomies and automated surgeries [61, 62]. The elastic (shear) modulus of the brain tissue was taken to be between 1 and 8 kPa, spanning the values found in the literature [31, 32, 63–66]. The radius of the simulated section of brain tissue was taken to be in the range of 100–200 µm, half of the typical distance between two penetrating arterioles in the mouse cortex [67, 68]. In the models where the deformability of the tissue is modelled, we saw that the pressure changes in the PVS can cause deformation in the brain and affect fluid flow in the PVS (Additional file 13: Fig S6). To model the pressure changes on the pial surface of the brain more accurately, we repeated our simulations where the flow resistance model at the pial opening of the PVS was replaced with a fluid filled SAS connected to the PVS over the brain surface (Additional file 7: Figures S5, Additional file 8: S8).

The arteriolar dilations caused by heartbeats and those generated in response to local neural activity have very different temporal dynamics and amplitudes. While cardiac pulsations are small in size, the arteriolar dilations that accompany increases in local neural activity are substantially larger and longer lasting. Heartbeat drives changes of 0.5–3% in the radius of pial arteries in mice [16]. These pulsations travel at a speed of 0.5–10 m/s along the arterial tree [69–71]. Mice have a heartrate of 7–14 Hz when they are unanaesthetized and freely behaving [72]. In contrast with arteriolar pulsations which occur at the heart rate, these neurally-induced arteriolar dilations take one to three seconds to peak and last for several seconds in response to a brief increase in neural activity. In response to increases in local neural activity, cerebral arterioles can dilate by 20% or more in non-anesthetized animals [51, 73–77]. These dilations induce blood flow changes that are the basis for the blood-oxygen-level dependent (BOLD), functional magnetic resonance imaging (fMRI) [78–81] signal. Neural activity-driven changes in arteriolar diameter take place at a nominal frequency range of 0.1–0.3 Hz [72].

In this study, we used finite element simulations to model fluid flow in the PVS and the deformation of the brain tissue. To make the calculations and interpretation of results simpler, we assume a cylindrically symmetric geometry with the centerline of the arteriole as the axis of symmetry (Fig. 1b).

## Results

### Part 1—directional CSF flow in the PVS

We first investigated the hypothesis that arteriolar wall movements (heartbeat-driven pulsations, functional hyperemia-driven dilations) could drive directed CSF flow. Both experimental [16–18, 82] and computational [19, 28, 83] studies have suggested the possibility of directed CSF flow as a result arteriolar wall movements, especially heartbeat pulsations. In our models, the space between the penetrating arteriole (the inner wall of the PVS) and the brain (the outer wall of the PVS) is filled with fluid. Fluid enters or exits the PVS through the SAS or the parenchyma (Fig. 1a). The flow resistance of the SAS was 0.01 times (or 0.1 times) the flow resistance of the PVS. The flow resistance of the parenchyma was 10 times that of the PVS. To quantify the flow driven by arteriolar wall movements alone, we imposed no pressure difference across the two ends of the PVS. We started our simulations with the assumption that the outer wall of the PVS was fixed (implying that the brain tissue is non-compliant), as has been done in other models [13, 19, 83]. The balance laws and boundary conditions used in the simulations are described in methods.

### Ignoring brain deformability leads to implausibly high pressures

**Fig. 2:**
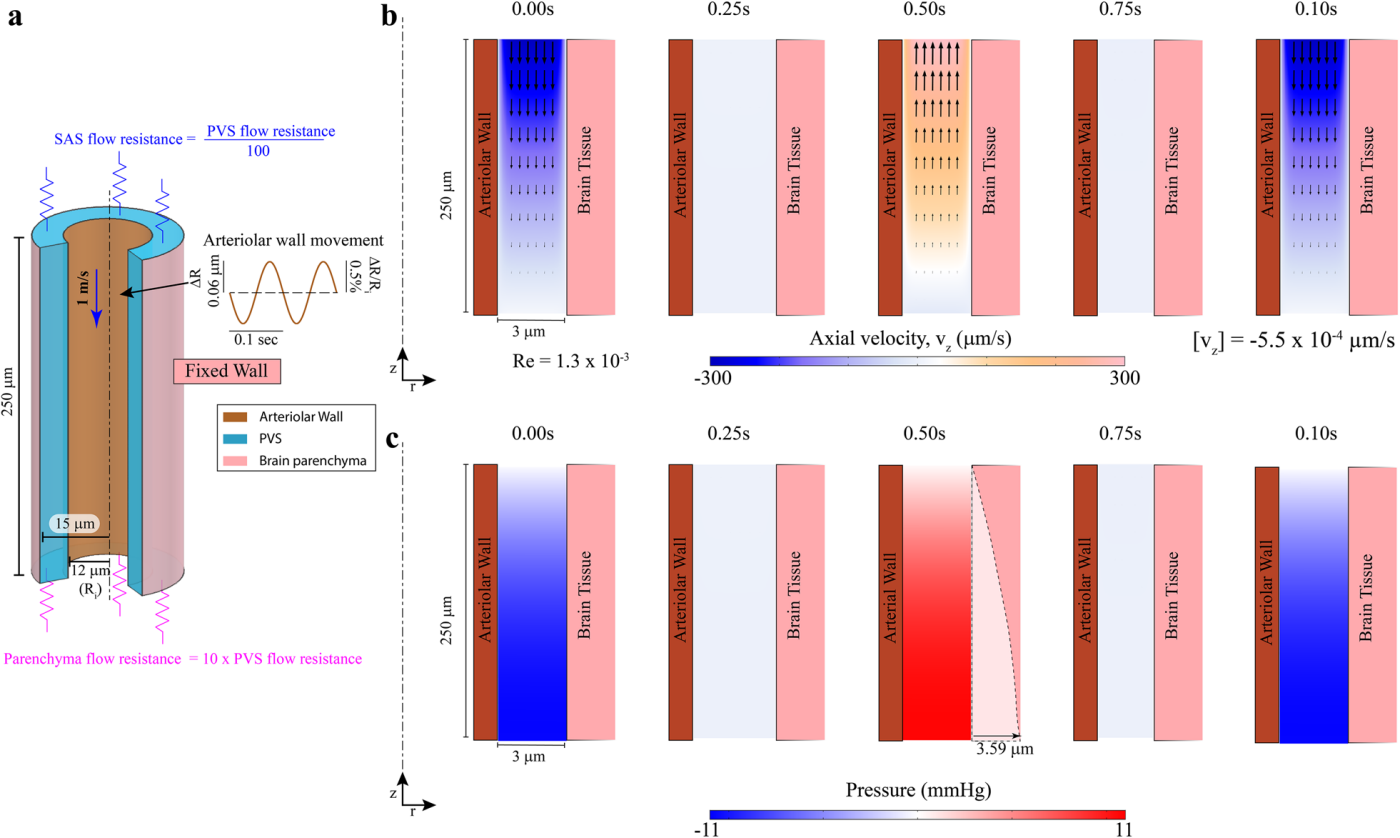
Modeling fluid flows and induced pressures while ignoring brain deformability. Note the geometry is depicted with an unequal aspect ratio in the radial (r) and axial (z) directions for viewing convenience. **a** Geometry of the PVS in in our model. The outer wall of the arteriole is shown in dark orange and the boundary of the brain parenchyma is shown in pink. The dashed line represents the centerline of the arteriole. The inset shows the imposed heartbeat-driven pulsations in arteriolar radius (± 0.5% of mean radius [16], R_i_) at 10 Hz, the heartrate of an un-anesthetized mouse. The pulse wave travels at 1 m per second along the arteriolar wall, into the brain [57, 58] (blue arrow). The flow through the SAS and the brain parenchyma was modelled by flow resistances (shown in blue and magenta respectively). In (**b**) and (**c**) a cross section of the PVS is shown together with the surrounding arteriolar wall (on the left) and brain tissue (on the right). **b** Plot of the fluid velocity induced in the PVS by the arteriolar pulsation. Contour showing the axial velocity (velocity in the z-direction) in a cross-section of the PVS. The colors indicate the direction and magnitude of flow. Fluid velocity vectors (arrows) are provided to help the reader interpret the flow direction from the colors. Heartbeat pulsations drive negligible unidirectional flow with a mean flow speed(-[v_z_]) of 5.5 × 10^−4^ µm/s. To make the arteriolar wall movements clearly visible, we scaled the displacements by a factor of 10 in post-processing. **c** Fluid pressure in the PVS corresponding to the flow shown in (**b**). Pressure changes due to fluid flow in the PVS reach several mmHg. These pressures will deform the soft brain tissue, which has a shear modulus of 1–8 kPa [63, 144] (8–60 mmHg). The dotted line shows the estimated deformation in the brain tissue (shear modulus 4 kPa–Kirchhoff/De Saint–Venant elasticity with Poisson ratio of 0.45) from the pressure shown in the figure. Under these assumptions, the deformations in the brain tissue are 60 times bigger (3.59 µm) in magnitude compared the peak of heartbeat driven pulsations (0.06 µm—shown on inset in (**a**)). Therefore, the deformability of brain tissue cannot be neglected

We investigated the fluid flow in the PVS (with a non-compliant brain) driven by heartbeat pulsations, the smaller of the two arteriolar wall motions considered in this study. To simulate the peristaltic wall motion of arterioles due to the heartbeat, the position of the inner wall of the PVS was prescribed via a travelling sinusoidal wave whose amplitude [16], frequency [72] and velocity [69, 70] were taken from experimental observations in mice. The results of the simulation with Darcy-Brinkman model are shown in Fig. 2 and Navier–Stokes model are shown in Additional file 2: Fig S1.

When the dimensions of the PVS in the simulations were of anatomically realistic size (3 µm wide and 250 µm long), we observed no appreciable net unidirectional movement of fluid. The average downstream velocity of fluid was 5.50 × 10^−4^ µm/s (1.84 × 10^−3^ µm/s for Navier–Stokes model) with an average flow rate of 0.14 µm^3^/s (0.47 µm^3^/s for Navier–Stokes). Instead of directional pumping, we saw periodic fluid movement in and out of PVS (Fig. 2b) with peak velocity magnitude in the range of 300 µm/s (Reynolds number, Re = 1.3 ×1 0^−3^), resulting in an oscillatory flow with negligible net unidirectional movement. We also repeated the simulation without the flow resistances (Additional file 3: Fig S2) and found an average downstream velocity of 2.95 × 10^−3^ µm/s. There was essentially no net fluid movement in these conditions because the wavelength of the cardiac pulsation (0.1 m in mice, see Table 1) is much longer than the PVS (150–300 µm). When the wavelength of the pulsation is substantially larger than the length of the PVS, the arteriolar wall movement cannot capture the shape of the peristaltic wave on the scale of cerebral arterioles. Effectively, the entire length of arteriolar wall moves in or out almost simultaneously. This effect can be better understood by comparing the arteriolar wall movement in a 250 µm arteriole (Additional file 3: Fig S2) with a 0.1 m arteriole (Additional file 4: Fig S3).

Our results are very similar, in terms of magnitude and direction of fluid velocities (Fig. 2b), to those obtained by Asgari et al. [13], who used a similar PVS geometry in their model. Asgari et al. [13] showed that large oscillatory fluid flow in the PVS can promote fluid mixing within the PVS and in between the PVS and the SAS and thus improve metabolite transport. When we simulated a PVS 0.1 m in length, we saw pumping of fluid, consistent with Wang and Olbricht [19], and Schley et al. [28] with an average downstream speed of 143.2 µm/s. However, these models predict pressure differences of up to 2.0 × 10^5^ mm of Hg (Additional file 4: Fig S3b). This is comparable to the pressures found on the ocean seabed, under several kilometers of water (2.0 × 10^5^ mm of Hg = 2.7 km of water), which is physically implausible. Our model does not consider the asymmetric time course of the heartbeat pulsation waveform, the non-circular shape of the PVS or the PVS surrounding pial arteries [16, 17]. We addressed these questions in a previous study [84], where we showed that unphysiologically large amplitude pulsations (with a peak-to-peak diameter change of 50%) are required for appreciable pumping. Altering the PVS shape or waveform of the pulsation did not achieve directional pumping. Instead, these simulations [84] showed that directional CSF flow, as observed in experiments [16, 17], can be explained by very small (< 0.05 mm Hg) pressure differences in the system that could be naturally [85] occurring, or generated by the injections of the tracer [86, 87].

Models where the brain-PVS interface is fixed in position presumes that the brain tissue is non-compliant. This assumption is only valid if the pressures produced are small relative to the elastic modulus of the brain. When the brain is presumed to be non-compliant, our simulations show that the peak pressures in the PVS during pulsations can reach 11 mmHg (Fig. 2c) (0.32 mm Hg for Navier–Stokes). Given that the brain is a soft tissue with a shear modulus in the range of 1–8 kPa [29–32] (7–30 mmHg), we estimated that the peak displacement of the brain tissue induced by the pressure profile in Fig. 2c would be 3.59 µm (with a shear modulus of 4 kPa). The pressure profile for the Navier–Stokes model (Additional file 2: Fig S1b) predicts a displacement of 0.08 µm. This displacement cannot be ignored, because the arteriolar wall displacement driving the flow is only 0.06 µm. We conclude that pressures induced by the flow demand that the mechanical properties of brain tissue and its deformability must be accounted for to accurately simulate fluid dynamics.

### Arteriolar wall motions cannot drive directed fluid flow in the PVS

**Fig. 3:**
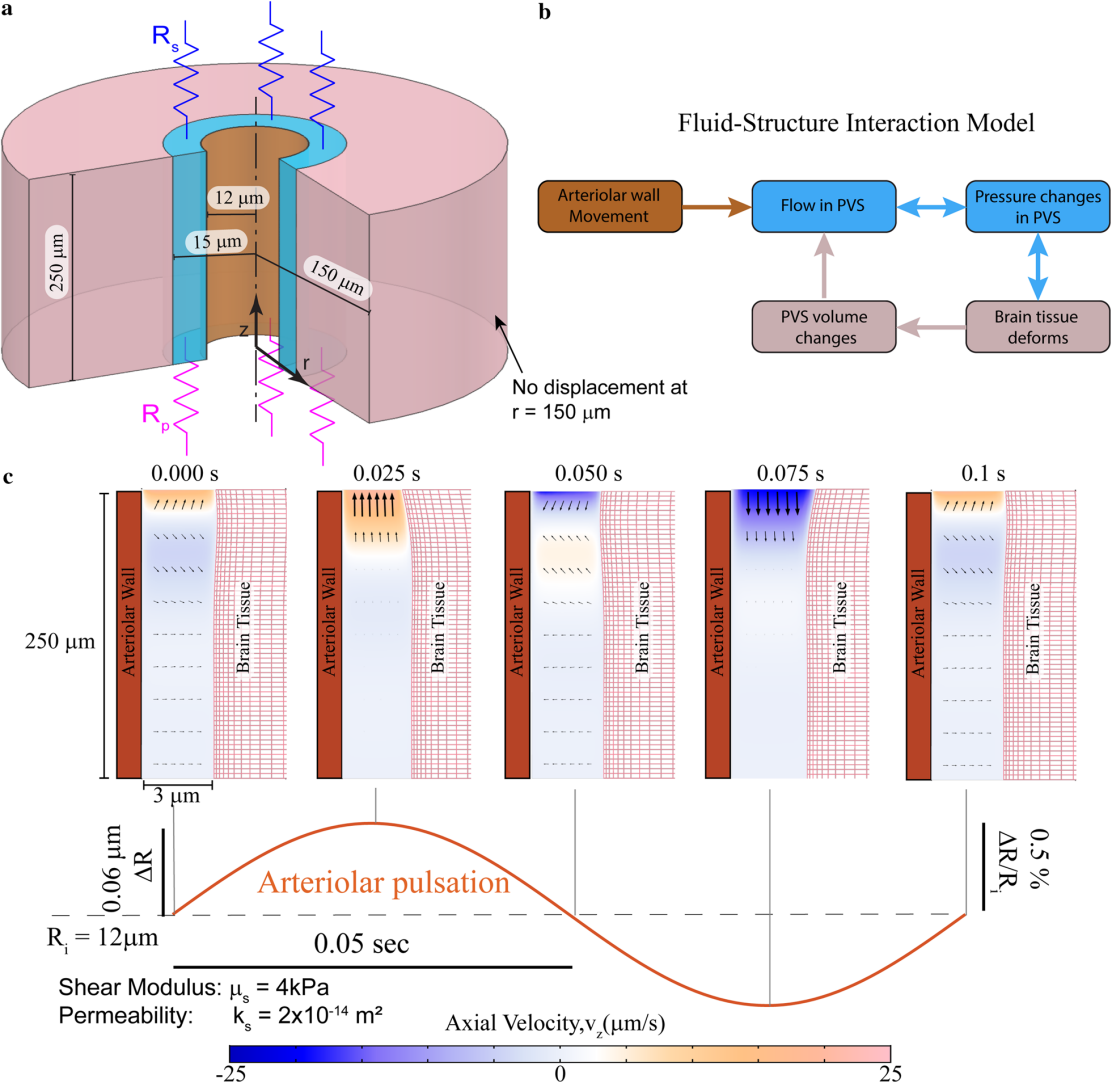
Arteriolar pulsation-driven flow in the PVS in an arteriolar-brain model with realistic mechanical properties. Note the geometry is depicted with an unequal aspect ratio in the radial (r) and axial (z) directions for viewing convenience. **a** The model of the penetrating arteriole. The brain tissue is modelled as a compliant solid. The subarachnoid space is modelled as a flow resistance (R_s_) at the pial end of the PVS and the parenchyma is modelled as a flow resistance (R_p_) at the other end. For the simulation with the subarachnoid space modelled as a fluid filled region, see Additional file 7: Fig S5. **b** A schematic depicting the fluid–structure interaction model described in (**a**). The arteriolar wall movement drives the fluid movement in the PVS. This fluid movement is coupled with the pressure changes. These pressure changes deform the brain tissue, changing the shape and volume of the PVS. These volume changes will affect the flow in the PVS, as demonstrated in (**c**). **c** Plot showing the axial fluid velocity (velocity in the z-direction) in a cross section of the PVS, when the arteriolar wall movement is given by periodic pulsations. The amplitude and frequency of the arteriolar pulsations are taken to be typical values for cerebral arterioles in mice. Fluid velocity vectors (arrows) are provided to help the reader interpret the flow direction from the colors. The region in white has little to no flow. These plots show that there is no appreciable flow into the PVS driven by arteriolar pulsations. Note: Arteriolar and brain tissue displacements induced by arteriolar pulsations are very small (< 0.1 µm). To make the movements clearly visible, we scaled the displacements by a factor of 10 in post-processing. These calculations were performed with fluid permeability, k_s_ = 2 × 10^−14^ m^2^ and tissue shear modulus µ_s_ = 4 kPa

We modified our model by treating the brain as a compliant, elastic solid (Fig. 3a). The pressure and the fluid shear forces in the PVS were coupled to the elastic deformation in the brain tissue using force-balance equations at the interface. We coupled the fluid velocity with the velocity of deforming brain tissue, to create a fully-coupled, fluid–structure interaction model (Fig. 3b). In this model, the pressure changes in the PVS directly affect the deformation of the brain tissue and have a feedback effect on the flow in the PVS. The balance laws and boundary conditions used in this problem are described in the methods.

We investigated how a compliant brain tissue model would respond to arteriolar pulsations. We imposed movement of the arteriolar wall with the same dynamics used in our previous model and visualized the resulting fluid flow in the axial direction (v_z_) (Fig. 3c). Throughout the pulsation cycle, most of the fluid in the PVS showed little to no movement (white). The flow observed in these simulations has a Reynolds number of 1.14 × 10^−4^. The average downstream velocity of fluid was 2.6 × 10^−3^ µm/s.

**Fig. 4:**
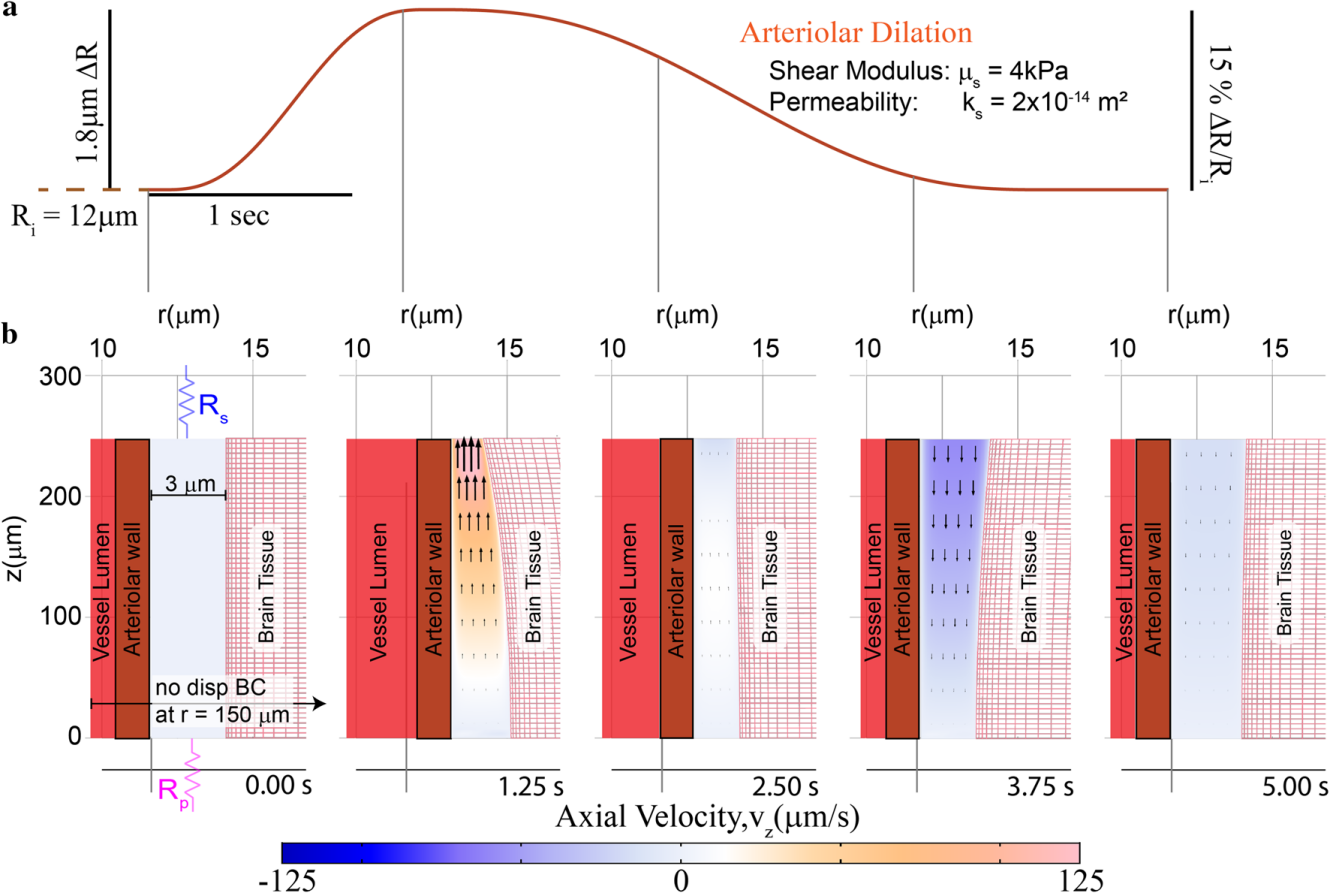
Functional hyperemia-driven flow in the PVS in an arteriolar-brain model with realistic mechanical properties. Note the geometry is depicted with an unequal aspect ratio in the radial (r) and axial (z) directions for viewing convenience. **a** Plot of the prescribed arteriolar wall movement for functional hyperemia. All the other boundary conditions used in this simulation are similar to the ones shown in Fig. 3a. **b** Contours showing the axial velocity (velocity in the z-direction) in a cross section of the PVS, when the arteriolar wall movement is given by a typical neural activity-driven vasodilation response. The boundary conditions (shown in the left panel) for this simulation are the same as the ones shown in Fig. 3. Compared to heartbeat-driven pulsations (Fig. 3c), vasodilation-driven fluid flow occurs through the entire length of the PVS and has substantially higher flow velocities. The model also predicts that the vasodilation can also cause significant deformation in the brain tissue. A portion of the vessel lumen is shown in red to provide a sense of vasodilation. These calculations were performed with fluid permeability, k_s_ = 2 × 10^−14^ m^2^ and tissue shear modulus µ_s_ = 4 kPa

To study the fluid flow in the PVS driven by functional hyperemia, we imposed arteriolar wall motion in our model that matched those observed in awake mice during a typical functional hyperemic event [50, 51, 77] (Fig. 4a). The mathematical formulation of this problem was identical to the previous simulation, with the exception that the arteriolar wall movement was given by a typical vasodilation profile instead of a heartbeat-driven peristaltic wave (Fig. 4a). Compared to the flow driven by arteriolar pulsations, functional hyperemia-driven flow in the PVS had substantially higher flow velocities (Fig. 4b). The flow observed in these simulations has a Reynolds number of 4.15 × 10^−4^. However, the average downstream velocity of fluid (over 10 s) was 0.12 µm/s.

There was very little directional fluid flow in the PVS due to arteriolar wall motions. The average downstream velocity of fluid driven by pulsations and hyperemia in the PVS remained less than 1 µm/s over a wide range of assumptions and parameters. Changing the brain tissue model from nearly incompressible (Poisson’s ratio of 0.45) to a completely incompressible (Poisson’s ratio of 0.5), Neo-Hookean model (Additional file 5: Fig S4, Additional file 6: S7) had minimal impact on the fluid velocities. Simulations where the subarachnoid space (SAS) was modeled as a fluid-filled region connected to the PVS (Additional file 7: Fig S5, Additional file 8: S8) had similar flow velocities. We also calculated directional fluid flow driven by pulsations and hyperemia with different values of PVS width, permeability and shear modulus of the brain tissue. When the flow in the PVS was modeled by Navier–Stokes equation, the average downstream velocity of fluid was 0.078 µm/s per heartbeat cycle and 0.16 µm/s for 10 s of functional hyperemia. Changing the flow resistance of the SAS from 0.01 times the resistance of the PVS to 0.1 times the PVS resistance further reduced the average downstream flow. The highest average downstream fluid velocity of 0.16 µm/s was obtained when the arteriolar wall motion was like hyperemia, the width of the PVS was 3 µm, and the fluid flow was modeled by Navier–Stokes equations in a brain tissue with elastic modulus of 8 kPa. This average downstream velocity of 0.16 µm/s is two orders of magnitude smaller than the experimentally observed downstream velocities in the PVS of ~ 20 µm/s [16, 17]. Our simulations suggest that the arteriolar pulsations and dilations cannot drive directed, net CSF flow into the PVS.

### Part 2—fluid exchange between the PVS and the SAS

Our simulations suggested that the directional flow driven by arteriolar pulsations and functional hyperemia is negligible. Therefore, we considered a different paradigm of metabolite clearance from the PVS, fluid exchange between the PVS and the SAS. Here, we use the well-established CSF flow through the SAS [1, 2, 5–7, 20–22, 26, 27] as the basis for metabolite clearance from the PVS. We propose that the fluid exchanged between the PVS and the SAS could be carried away by the existing directional flow in the SAS, thus aiding the clearance of metabolites from the PVS. This assumption is similar to the fixed-concentration boundary condition used at the SAS-PVS interface in studies proposing dispersion as a mechanism for clearance of metabolites [13]. In order to quantify the fluid exchanged between the PVS and SAS, we calculated the volume exchange fraction, Q_f_, driven by arteriolar wall movement. The volume exchange fraction was defined as the ratio of the maximum amount of fluid leaving the PVS to the total volume of fluid in the PVS. The volume exchange fraction is an indicator of the total volume change of the PVS (see Additional file 1: Appendix for full mathematical definition). We use the volume exchange fraction as the metric for the fluid exchange between the SAS and the PVS and metabolite clearance from the PVS. The transfer of metabolites from the brain interstitial space to the PVS is not explicitly modelled here, and is assumed to occur via diffusion [10, 12, 14, 24, 25].

### Functional hyperemia but not pulsation drives appreciable fluid exchange

**Fig. 5:**
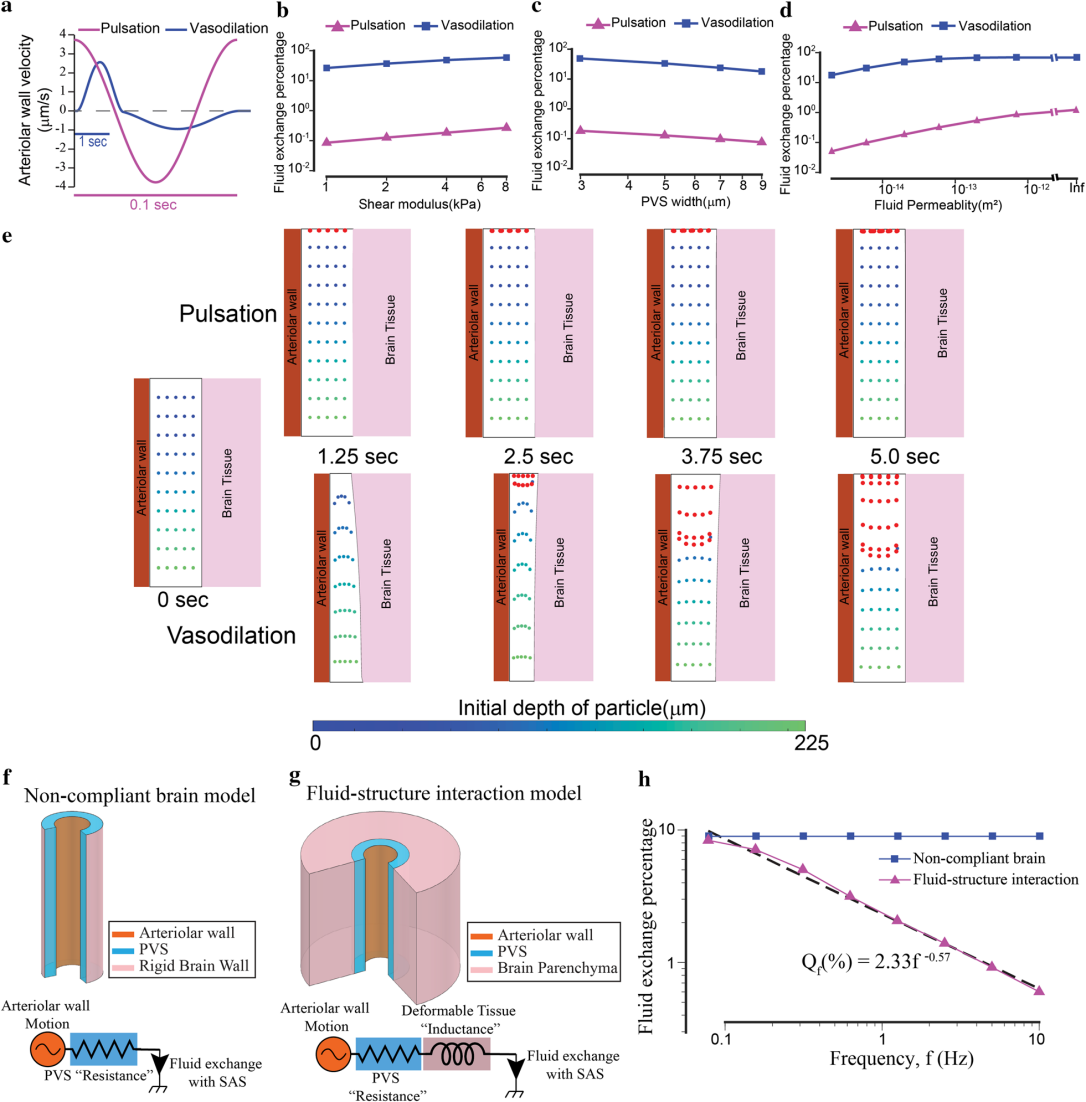
Functional hyperemia but not arteriolar pulsation drives appreciable fluid exchange between the PVS and the SAS. The difference in the fluid exchange driven by the two mechanisms is because of the deformability of brain tissue. **a** The arteriolar wall velocities induced by pulsations and hyperemia used in our simulations are similar in magnitude. The time scales are different for pulsations and hyperemia. **b**–**d** Vasodilation drives two orders of magnitude higher fluid exchange between the PVS and subarachnoid space compared to heartbeat driven pulsations. The plots show the changes in fluid exchange percentage, the percentage of fluid in the PVS exchanged with the SAS, with change of model parameters. The model predicts that compared to arteriolar pulsations, the vasodilation driven fluid exchange percentage is two orders of magnitude higher. This difference is similar for different values of elastic modulus of the brain (**b**), the width of the PVS (**c**) and the fluid permeability of the PVS (**d**). In (**d**), when the permeability is infinite, Darcy-Brinkman’s law transforms into Navier–Stokes’ law for fluid flow. All the plots are made on a log–log scale because the parameters were changed by 1–3 orders of magnitude. **e** Comparison of particle motion in the fluid of the PVS during arteriolar pulsations and vasodilation. The blue-green dots represent fluid in the PVS, with the colormap showing the initial position (depth) of the fluid particle in the PVS. Fluid particles near the SAS (red dots) are added once every 0.5 s to the simulation to simulate fluid mixing between the PVS and the SAS. There is very little fluid movement driven by arteriolar pulsations. Vasodilation drives appreciable fluid exchange between the PVS and the SAS. These calculations were performed with fluid permeability, k_s_ = 2 × 10^−14^ m^2^ and tissue shear modulus µ_s_ = 4 kPa. **f** Geometry for a model in which the brain is a rigid boundary to the PVS (top) and the equivalent circuit diagram (bottom). The driver for fluid flow is the arteriolar wall motion. The flow resistance of the PVS can be modelled by a simple resistor is independent of the frequency of the arteriolar wall movement. **g** Geometry for the fluid–structure interaction model with a deformable brain (top) and the equivalent circuit diagram (bottom). The driver for fluid flow is the arteriolar wall motion. The total flow resistance of the system can be modelled by a resistance from the PVS and an inductance because of the deformable tissue. In this model, the flow resistance of the system increases with increase in the frequency of the arteriolar wall motion. This means that for arteriolar wall motion at high frequency, less fluid will be exchanged between the PVS and the SAS. **h** Plot shows the relation between fluid exchange percentage and frequency of arteriolar wall motion. The arteriolar wall motion was given by a 4% peak-peak sinusoidal wave with different frequency values. The default values were used for all other parameters (see Table 1). For very low frequencies (< 0.1 Hz), the fluid exchange driven by the arteriolar wall is same whether or not brain deformability is taken into account. For higher frequencies, the fluid exchange percentage has an inverse power law relation with the frequency of arteriolar wall motion

We measured the fluid exchange between the PVS and the SAS driven by the arteriolar pulsations and functional hyperemia from the models presented in the previous section. For the default parameters (Table 1), arteriolar pulsations driven by heartbeat cause a mere 0.21% (Q_f_ = 0.0021) of the fluid in the PVS to be exchanged with the SAS and the parenchyma per cardiac cycle. For the same parameters, a single brief hyperemic event could exchange nearly half (Q_f_ = 0.4946) of the fluid in the PVS with the SAS. This difference in the fluid exchange driven by pulsations and hyperemia can be inferred from the fluid velocities in the PVS, shown in Figs. 3 and 4. The differences in flow velocities resulting from the two arteriolar wall motions are very interesting, considering that the arteriolar wall velocity from both pulsations and hyperemia are of the same order (Fig. 5a). The small flows in the PVS driven by pulsations were due to the compliance of the brain, as any pressure gradient that could generate substantial fluid movement will be dissipated on deforming the brain tissue instead. This result is in contrast to the calculations of Asgari et al. [13], which suggested that the pulsatile flow in the PVS could improve metabolite clearance through dispersion. The relatively large pulsatile velocities calculated by Asgari et al. [13], in the range of 120 µm/s (as opposed to our calculations of less than 25 µm/s) can be attributed to not considering the elastic response of the brain tissue.

We calculated the fluid exchange fraction for different values of shear modulus of the brain tissue (Fig. 5b), and width (Fig. 5c) and permeability (Fig. 5d) of the PVS. For all the tested parameters, functional hyperemia-like dilations drove substantial fluid movement in the PVS. Compared to arteriolar pulsations, the vasodilation-driven fluid exchange between PVS and SAS was two orders of magnitude higher under a wide range of model parameters (Fig. 5b–d). When the fluid is modeled by the Navier–Stokes equations (infinite permeability in Fig. 5d), 69.8% of fluid in the PVS is exchanged with the SAS for a single, brief hyperemic event, whereas arterial pulsations only caused 1.37% of the fluid in the PVS to exchange with the SAS per cycle. Changing the flow resistance of the SAS from 1/100th to 1/10th of the resistance of the PVS had a minimal effect on the fluid exchange fraction. For example, in simulations where the SAS flow resistance was replaced by 1/10th of the PVS resistance (instead of 1/100th), for the default parameters (see Table 1) one pulsation cycle drove fluid exchange of 0.13% (instead of 0.21%) and a single hyperemic event drove a fluid exchange of 48.0% (instead of 49.5%). This effect of changing the SAS flow resistance on the fluid exchange was much smaller compared to other parameters of interest (Fig. 5b–d).

To understand the flow near the brain surface and into the PVS, we define two Péclet numbers, Pe_0_ and Pe_50_, near the surface of the brain (z = La) and 50 µm below the surface (z = La-50 µm) of the brain respectively (see “Methods”). For the default parameters, the values of Pe_0_ and Pe_50_ are 0.82 and 0.19 respectively for pulsation driven flow, confirming that transport in the PVS away from the surface of the brain appears to be diffusion-dominated. The values of Pe_0_ and Pe_50_ for functional hyperemia-driven flow are 2.97 and 1.96 respectively, showing that the fluid exchange caused by vasodilation can improve metabolite clearance compared to diffusion.

### Fluid exchange by arteriolar pulsations is not compounded over time

Arteriolar pulsations occur at nearly two orders of magnitude higher frequency compared to functional hyperemia. Pulsations and hyperemia occur nominally 10 Hz and 0.2 Hz, respectively. If fluid exchange by arteriolar pulsations compounded with time, the fluid exchange between the PVS and the SAS be similar for arteriolar pulsations and functional hyperemia over equal time periods. To test this possibility, we calculated fluid particle trajectories in the deforming geometry of the PVS (see Additional file 1: Appendix for full mathematical description of boundary value problem for particle tracking in a deforming domain). The blue-green dots in Fig. 5e represent fluid in the PVS, with the colormap showing the initial position (depth) of the fluid particle in the PVS. Fluid particles near the SAS (red dots) are added once every 0.5 s to the calculation to simulate the possibility of fluid exchange between the PVS and the SAS. The results of these calculations indicate that a single hyperemic event can cause substantially more fluid movement in the PVS compared to arteriolar pulsations over the same time (Fig. 5e, also see videos Additional file 10: SV1 and Additional file 11: SV2). These calculations suggest that when the flow in the PVS is modeled with coupled soft brain tissue mechanics, functional hyperemia can drive appreciably higher fluid exchange between the PVS and the SAS as compared to arteriolar pulsations over the same time period.

We compared the fluid particle trajectories for arteriolar pulsations and dilations only for a short 5 s time interval. This might not be a fair comparison because functional hyperemia might only occur occasionally, while heartbeat pulsations are perpetually present. Calculating fluid trajectories over larger time periods than what we showed here (5 s), while keeping the time step small enough to capture the details of peristaltic wave (pulsations traveling at 1 m/s would traverse an arteriole of length 250 µm in 2.5 × 10^−4^ s) is challenging as the accuracy of longer simulations would be severely affected by the accumulation of numerical errors. Estimates of slow fluid drift from oscillatory flows can be obtained by semi-analytical methods [88] and by building representative experimental systems [89]. However, our simulations suggest that there is almost no oscillatory flow in the PVS of penetrating arterioles. This is demonstrated by the majority of the PVS shown covered in white (indicating that there is no flow) in Fig. 3c.

### Brain tissue deformability affects fluid flow in the PVS

**Fig. 6:**
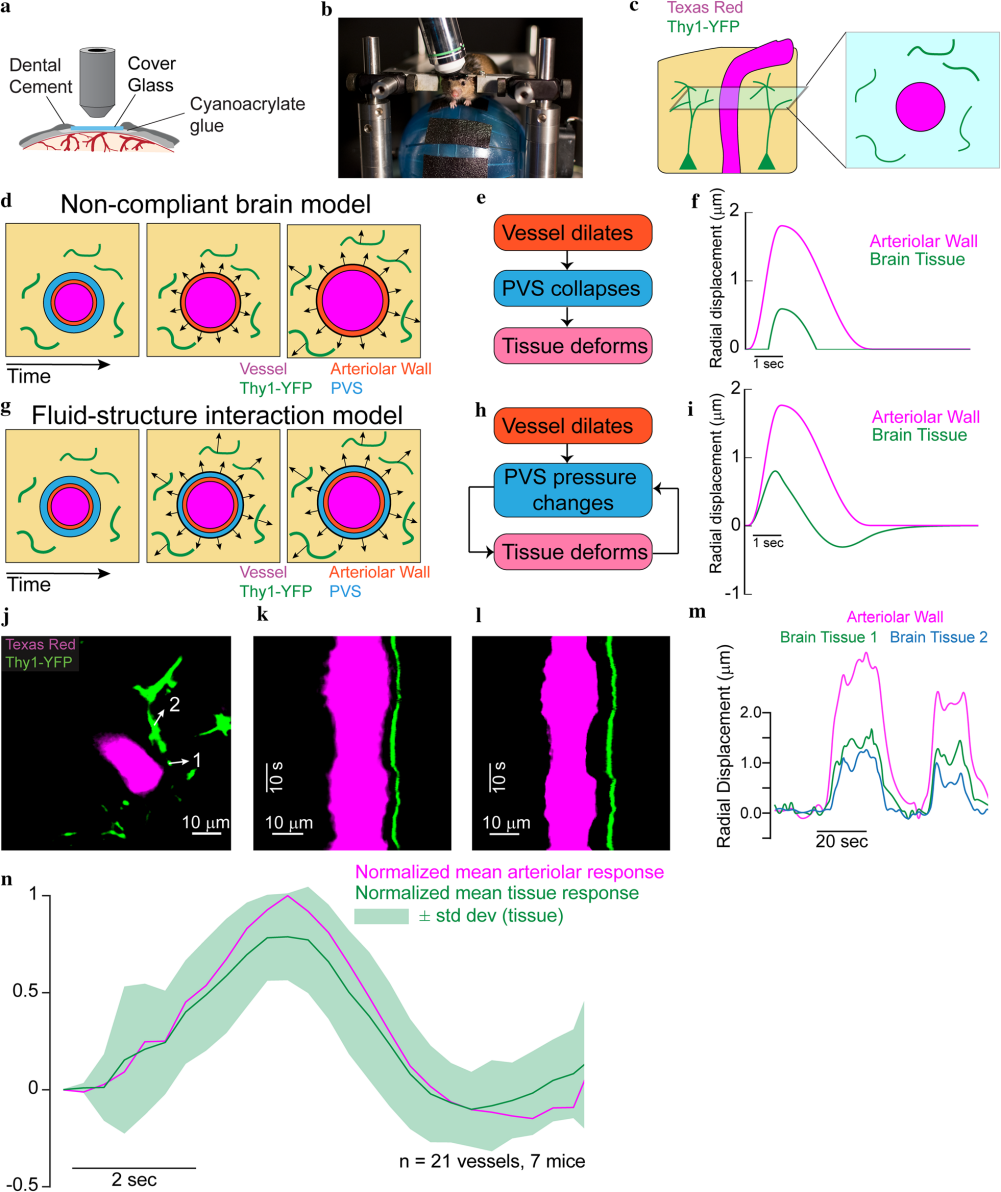
In-vivo measurement of brain tissue-displacement suggests that the brain tissue can deform because of pressure changes in the PVS. **a** Schematic of a thin skulled window. Mice implanted with a thinned-skull window (PoRTS window [49]) were imaged under a two-photon laser scanning microscope (2PLSM). The mice were head-fixed and allowed to run voluntarily on a spherical treadmill. **b** Experimental setup for two-photon microscopy. Mice were head-fixed and placed on a spherical treadmill. **c** Schematic of the fluorescent elements in the brain parenchyma (left) surrounding a penetrating arteriole and the expected 2-D images under a 2PLSM (right). A retro-orbital injection of Texas red dye conjugated dextran (40 kDa, 2.5% w/v) makes the vessel lumen fluorescent. The yellow fluorescent protein is expressed by a sparse subset of neuronal processes. **d** A schematic of the brain tissue deformations expected when pressure changes in the PVS do not deform the brain until PVS collapse. The position of the vessel wall and the PVS are shown on the left. When the arteriole dilates, the brain tissue would not deform until the PVS completely collapses (middle). After the PVS has collapsed completely, the brain tissue would start deforming(right). **e** Flow chart of the mechanism of brain tissue deformation in a “non-compliant brain” model. **f** The expected radial displacement in the brain tissue in response to arteriolar dilation when deformability of the brain is initially ignored. The brain tissue does not deform until the PVS has completely collapsed. Note that the expected values are based on the displacement used for our simulations and actual values may vary. **g** A schematic of the expected brain tissue deformation from a fluid–structure interaction model. Here the pressure changes in the PVS cause the brain tissue to deform. **h** Flow chart of the mechanism of brain tissue deformation in a fluid–structure interaction model. **i** The expected radial displacement in the brain tissue in response to arteriolar dilation in the fluid–structure interaction model (also see Additional file 13: Fig S6). Note that the expected values are based on the displacement used for our simulations and actual values may vary. **j** Median frame of the 2D image collected during in vivo imaging. Example image of penetrating arteriole (magenta) and YFP expressing neurons(green). The arrows show the direction of the displacement measured at the location indicated by the tail of the arrow. **k**, **l** Projection in time along a line running through the arrows 1 and 2 respectively shown in (**j**). The images show that when the vessel dilates (indicated by a widening of the vessel in magenta), there is a corresponding radially-outward deformation in the brain tissue (indicated by the movement of the green line). Time moves forward in the in the vertically downward direction in both images. **m** The calculated radial displacement in the brain tissue in response to changes in arteriolar radius. The data suggests that the brain tissue deforms due to pressure changes in the PVS before the PVS completely collapses. **n** The average (7 mice, 21 vessels) peak-normalized impulse response of the radial displacement of the arteriolar wall (magenta) compared to the average peak-normalized impulse response of the radial displacement in the brain tissue (only one data point per vessel was used for this calculation). The data shows that there is no delay between displacement of arteriolar wall and the tissue, suggesting that the brain tissue deforms due to pressure changes in the PVS as predicted by the fluid–structure interaction model

There are two main reasons why functional hyperemia drives large fluid exchange between the PVS and the SAS, while arteriolar pulsations are ineffective at driving fluid movement in the PVS. Firstly, heartbeat-driven changes in arteriolar diameter are very small (0.5–4% [16]) in magnitude compared to neural activity-driven vasodilation (10–40% [51]) and therefore there is a large difference in the volume of fluid displaced by the two mechanisms. Our measurements in vivo also confirmed that the diameter changes driven by heartbeat (Additional file 9: Fig S9) are in the 0.5–4% range while the diameter changes driven by vasodilation are in the 10–40% range (Fig. 6m). A difference in the magnitude of blood volume change driven by heartbeat and hyperemia has also been observed in macaques [90] and humans [91] using functional magnetic resonance imaging (fMRI). Secondly, there is a large difference in the frequency of pulsations (7–14 Hz [72] in mice, nominally 1 Hz in humans) and hyperemic (0.1–0.3 Hz [77, 92]) motions of arteriolar walls. Fast (high frequency) movement of arteriolar walls cause larger changes in pressure, which will deform the brain tissue rather than driving fluid flow. Also, deformable (elastic) elements absorb more energy at higher frequencies. If the electrical circuit equivalent of flow through the PVS while ignoring brain deformation is analogous to a resistor, the equivalent of flow through the PVS with a deformable brain is analogous to a resistor and inductor in series (Fig. 5f–g). In other words, arteriolar wall motion at higher frequencies drives less fluid movement compared to arteriolar wall movement at lower frequencies. A similar phenomenon has been studied extensively in the context of blood flow through deformable arteries and veins [93–96]. We compared the fluid exchange percentage for an arteriolar wall movement given by a sine wave (4% peak to peak) of different frequencies, and found that the fluid exchange percentage showed an inverse power law relationship to the frequency of the pulsation (f) ($Q_{f} = 2.33f^{ - 0.57} \%$ for the default parameters, Fig. 5h). These calculations show that slow frequency arteriolar motions can drive better fluid exchange between the SAS and the PVS, when the PVS is surrounded by a deformable brain tissue.

### Part 3—measurement of brain tissue deformation in vivo

One of the main predictions of the fluid–structure interaction model is that there will be deformation of the soft brain tissue in response to the pressure changes in the PVS driven by arteriolar dilation. The simulations suggested that the pressure changes in the PVS due to this flow will deform the brain tissue by up to 1.2 µm for an arteriolar dilation of 1.8 µm (Additional file 13: Fig S6). To test this prediction, we measured displacement of the cortical brain tissue surrounding penetrating arterioles in awake, head-fixed B6.Cg-Tg(Thy1-YFP)16Jrs/J (Jackson Laboratory) mice [97] using two-photon laser scanning microscopy [50]. These transgenic mice express yellow fluorescent protein (YFP) in a sparse subset of pyramidal neurons, rendering their axons and highly fluorescent [98]. Mice were implanted with polished, reinforced thinned-skull windows [49] (Fig. 6a) to avoid inflammation [99], disruption of mechanical properties [100] and the hemodynamic and metabolic effects [101] associated with craniotomies. We simultaneously imaged processes of Thy1-expressing neurons and blood vessel diameters (labeled via intravenous injection of Texas-red dextran) (Fig. 6b). Arterioles in the somatosensory cortex dilate during spontaneous locomotion events due to increases in local neural activity [77], so we imaged these vessels that normally undergo large vasodilations in the awake animal. We performed piecewise, iterative motion correction of the images relative to the center of the arteriole (see Methods) in order to robustly measure the displacement of brain tissue during arteriolar dilations. We visually verified the measured brain tissue displacements.

We considered two possible paradigms of brain deformation, a “non-compliant brain” model and a fluid–structure interaction model. We predict the two paradigms to yield completely different results in terms of the displacement of the brain tissue observed in vivo. In the non-compliant brain model, the brain tissue will be unaffected by pressure changes in the PVS. In this model, pulsations and small dilations of arterioles would cause flow in the PVS but no displacement of the brain tissue (Fig. 6c). Only after the arteriolar wall comes in contact with the brain tissue (and the PVS has fully collapsed), arteriolar dilation would cause tissue displacement (Fig. 6d). Therefore, displacement in the brain tissue in this model would be either non-existent (for small dilations), or similar to a “trimmed” version of the displacement of the arteriolar wall (Fig. 6e). Alternatively, in the fluid–structure interaction model, any movement of the arteriolar wall that can drive fluid flow in the PVS will result in pressure changes in the PVS that are sufficient to deform the ‘soft’ brain tissue, as predicted by our simulations (Fig. 6f, g). Therefore, displacement should be observed in the brain tissue as soon as the arteriolar wall starts to dilate. In the fluid–structure interaction model, the radial displacement in the brain tissue would be a scaled version of the radial displacement of the arteriolar wall (Fig. 6h).

### In-vivo brain tissue deformation is consistent with a fluid–structure interaction model

We calculated the radial displacement of the arteriolar wall and the brain tissue in vivo (n = 21 vessels, 7 mice) using two-photon microscopy. The radial displacement of the brain tissue was between 20 and 80% of the radial displacement of the arteriolar wall. The simulations suggest that such a variation is to be expected due to heterogeneity in the width and depth of the PVS and variations in the distance of the plane of imaging from the surface of the brain (Additional file 13: Fig S6a and S6b). Despite the variation in the amplitude of displacement in the tissue, our simulations predict that the peak-normalized displacement response of the brain tissue should have the same temporal dynamics as the arterial dilation (Additional file 13: Fig S6c). We used this result from the simulation to test the predictions of the model experimentally. We calculated the peak normalized impulse response of the displacements to locomotion (Fig. 6n). The calculations of tissue displacement for each arteriole (an example is shown in Fig. 6j–m), as well as the normalized impulse response for the brain tissue (Fig. 6n) suggest that the displacement in the brain tissue started as soon as the arteriolar dilations started. This implies that the brain tissue can deform due to pressure changes in the PVS, as predicted by the fluid–structure interaction model. Note that all the displacement values in the brain tissue used for calculating the average waveform reported in Fig. 6n were subject to a rigorous set of tests (see “Methods”) to account for motion artifacts. To visualize the brain tissue displacements accompanying vasodilation, we plotted a kymogram taken along diameter line bisecting the arteriole and crossing neural processes (Fig. 6k, l). Distance from the center of the arteriole is on the x-axis and time on the y-axis. Dilations appear as a widening of the vessel, while displacements of the brain tissue will show up as shifts on the x axis. This visualization was used as an additional step in validating the displacement values calculated by our method. For calculating the average waveform of tissue displacement shown in Fig. 6n, only one of the calculated displacement values per vessel that could also be visually verified was used. The displacement of the brain tissue is also apparent from visualizing the data. Additional file 14: Video SV3 shows 50 s of imaging data, where we can observe how the brain tissue (green) deforms in response to dilation of the vessel (magenta).

Interestingly, the fluid–structure interaction model predicted a negative radial displacement in the brain tissue, when the arteriole constricts or returns to its original diameter, which we did not observe. This anomaly can be explained by the fact that the fluid–structure interaction model neglects the elastic forces in the connective tissue (extracellular matrix) in the PVS. The PVS contains collagen fibers and fibroblasts, that are continuous with the extracellular space of the surrounding tissue [102, 103]. Collagen networks can have a highly non-linear elastic response when the loading is changed from compression to tension, and exhibit hysteresis during large, cyclic deformations [104, 105]. The elastic modulus of fibrous networks under tension can be 2–3 orders of magnitude higher than the elastic modulus in compression [106–108]. Connective tissue is made up of networks of fibers, and the energy cost of bending these fibers is several orders of magnitude smaller than stretching them. When the arteriole dilates, these fibers are subject to a compressive loading and they buckle (bend) rather than compress, and as a result generate very little elastic forces (Additional file 12: Fig S10b). However, when the arteriole constricts, these fibers are subjected to a tensile load (Additional file 12: Fig S10c) and produce significantly higher (2–3 orders of magnitude higher) elastic forces. However, our model only considers the fluid-dynamic forces in the PVS and neglects the elastic forces. This is one of the shortcomings of our model, that can be corrected in the future using models of poroelasticity [109–111] so as to account for the mutual interaction of flow and deformation within the PVS. Alternatively, the predicted negative radial displacement might be an artifact of modelling the brain tissue with a Poisson’s ratio of 0.45–0.5. While this range of Poisson’s ratio might be adequate to simulate the elastic behavior of the brain under compression, the brain behaves like a solid with a Poisson’s ratio of 0.3 under tension [60].

## Discussion

A summary of the model parameters and main results of all the simulations in this article are shown in Table 2 below. The pressure changes observed in the PVS, while assuming the brain tissue is non-compliant can cause appreciable deformation in the brain. For all the fluid–structure interaction models, the percentage of PVS fluid exchanged with the SAS is the main metric for metabolite clearance. The mean downstream speed (net directional flow velocity into the parenchyma) is negligible for all cases with realistic length of the arteriole.

While there have been several models investigating the fluid mechanics in the PVS [13, 19, 28], to our knowledge, none of them considered the impact of the soft, deformable brain tissue on CSF flow in the PVS. Our simulations show that fluid flow in a porous PVS resulting from the movement of the vessel walls can be affected by the deformability of the brain tissue. We have also presented empirical evidence to support our claim that the brain tissue deforms in response to pressure changes in the PVS. As far as we know, this is the first study to include deformability of the brain tissue in modeling fluid flow in the PVS and to use experiments to support the predictions of fluid dynamic simulations.

**Table 2:** Summary of Simulations

Figure	Brain model	Fluid permeability (m^2^)	Arteriolar wall movement	Mean downstream speed (µm/s)	PVS fluid exchanged with SAS	Conclusion
2	Non-compliant	2 × 10^−14^	Heartbeat ∆R_max_ = 0.06 µm	5.5 × 10^−4^	–	Expected brain deformation, 3.59 µm > pulsation amplitude, 0.06 µm
3	SVK, µ_s_ = 4 kPa (compressible)	2 × 10^−14^	Heartbeat ∆R_max_ = 0.06 µm	2.6 × 10^−3^	0.21%	No appreciable fluid exchange
4	SVK, µ_s_ = 4 kPa (compressible)	2 × 10^−14^	Hyperemia ∆R_max_ = 1.8 µm	0.12	49.46%	Appreciable fluid exchange by hyperemia
5b	SVK, µ_s_ = 1–8 kPa	2 × 10^−14^	Heartbeat/Hyperemia ∆R_max_ = 0.06/1.8 µm	–	Heartbeat 0.09–0.29% Hyperemia 26.96–59.55%	Fluid exchange of hyperemia ≈ 200 × fluid exchange of heartbeat
5c	SVK, µ_s_ = 4 kPa	2 × 10^−14^ PVS width = 3-9 µm	Heartbeat/Hyperemia ∆R_max_ = 0.06/1.8 µm	–	Heartbeat 0.08–0.21% Hyperemia 17.88–49.46%	Fluid exchange of hyperemia ≈ 200 × fluid exchange of heartbeat
5d	SVK, µ_s_ = 4 kPa	2 × 10^−15^ –7 × 10^−13^ & ∞ (Navier–Stokes)	Heartbeat/Hyperemia ∆R_max_ = 0.06/1.8 µm	–	Heartbeat 0.06–1.37% Hyperemia 17.85–70.55%	Fluid exchange of hyperemia ≈ 200 × fluid exchange of heartbeat
5 h	SVK, µ_s_ = 4 kPa	2 × × 10^−14^	∆R_max_ = 0.24 µm Frequency, f = 0.05-10 Hz	–	$Q_{f} = 2.33f^{ - 0.57}$	Fluid exchange is inversely related to frequency of arteriolar wall movement
S1	Non-compliant	∞ (Navier–Stokes)	Heartbeat ∆R_max_ = 0.06 µm	1.8 × 10^−3^	–	Expected brain deformation, 0.08 µm > pulsation amplitude, 0.06 µm
S2	Non-compliant	2x10^−14^ No SAS/parenchyma flow resistances	Heartbeat ∆R_max_ = 0.06 µm	2.6 × 10^−3^	–	Expected brain deformation, 0.71 µm > pulsation amplitude, 0.06 µm
S3	Non-compliant	2 × 10^−14^ Arteriole length = 100 mm	Heartbeat ∆R_max_ = 0.06 µm	143.2	–	Flow accompanied by 200,000 mmHg pressure changes
S4	Neo-Hookean, µ_s_ = 4 kPa (incompressible)	2 × 10^−14^	Heartbea ∆R_max_ = 0.06 µm	1.1 × 10^−4^	0.18%	No appreciable fluid exchange
S5	SVK, µ_s_ = 4 kPa (compressible)	2 × 10^−14^ SAS geometry explicitly modeled	Heartbeat ∆R_max_ = 0.06 µm	2.6 × 10^−3^	0.23%	No appreciable fluid exchange
S6	SVK, µ_s_ = 4 kPa (compressible)	2 × 10^−14^	Hyperemia ∆R_max_ = 1.8 µm	0.12	49.46%	Tissue displacement has a typical waveform
S7	Neo-Hookean, µ_s_ = 4 kPa (Incompressible)	2 × 10^−14^	Hyperemia ∆R_max_ = 1.8 µm	0.13	50.92%	Appreciable fluid exchange
S8	SVK, µ_s_ = 4 kPa (compressible)	2 × 10^−14^ SAS geometry explicitly modeled	Hyperemia ∆R_max_ = 1.8 µm	0.12	49.66%	Appreciable fluid exchange

Our model has several limitations, and the results should be interpreted with these limitations in mind. We assumed a cylindrically symmetric geometry for our model. In reality, the PVS around penetrating vessels can be eccentric and elliptical [16]. An eccentric annular region with a pulsating inner wall can cause a slow drift in the fluid particles [89]. However, the drift caused by eccentricity is not unidirectional, and the bulk movement of the particles is much smaller compared to the oscillations [16, 17]. We did not explicitly model fluid flow within the brain parenchyma and the effect of the aquaporin-4 channels found on the astrocyte endfeet lining the brain-PVS interface [10]. The flow through the parenchyma was only simulated indirectly through the parenchymal flow resistance at one end of the PVS. This can be rectified in future studies by using models of poroelasticity [109–111], which simultaneously simulate fluid movement through the extracellular space and the deformations in the brain tissue. Some authors have used poroelastic models to predict that oscillatory flows can enhance metabolite transport in the brain through dispersion [112]. Poroelastic models can also explore the effect of static and dynamic occlusions to flow [113, 114], which our fluid–structure model does not simulate. We also ignore the elastic response of the collagen fibers in the PVS and only simulate the fluid dynamics in this region.

Our results are in agreement with the findings of several experimental studies [16, 17, 24, 56, 82, 115], though they cast their results in a new light. Several studies have used microspheres to visualize the CSF movement in the PVS around pial arteries [16, 17]. Similar to their results, our simulations suggest that CSF oscillates with the frequency of heartbeat driven pulsations near the surface of the brain (Fig. 3c, Additional file 4: Figures S3 and Additional file 5: S4). It can also be shown that larger arteriolar pulsations can cause larger oscillations in CSF flow, similar to the case of induced hypertension found by Mestre et al. [16]. In contrast to the conclusions of these particle tracking studies [16, 17], our simulations suggest that arteriolar pulsations do not provide a driving force for net unidirectional movement of CSF. Our results also agree with the findings that voluntary running, which increases neural activity [116, 117] and induces functional hyperemia [92, 118] in several regions of the brain, enhances penetration of tracers in the brain parenchyma, when injected into the cisterna magna [82]. The silencing of neural activity [119, 120] (and therefore vascular activity) by anesthetics [121, 122] could explain diminished penetration of tracers in the brain parenchyma, when injected into the cisterna magna under anesthesia [123]. The variability in results between groups may be influenced by anesthesia type and levels, both of which have large effects on the amplitude of the arteriolar dilations elicited during functional hyperemia [51]. Finally, brain-wide hyperemia observed during REM sleep [124] could explain improved tracer transport in the brain observed during sleep [115]. We would like to point out that our model suggests that functional hyperemia enhances transport from the SAS to the parenchyma (and vice versa) by fluid exchange through the PVS. We do not claim that functional hyperemia improves flow in the proposed “glymphatic” pathway [125], as we did not explicitly model flow through the brain extracellular space or the paravenous space.

Our results have implications for the development and treatment of CNS disorders and suggest that in addition to its other physiological roles, functional hyperemia may serve to improve transport into and out of the brain by driving exchange of fluid in the PVS with the SAS. Many studies support the idea that vascular dysfunction can be a precursor to neurodegenerative diseases [126–128]. Our simulations suggest a mechanistic relation between neurovascular coupling and metabolite clearance from the brain, which could explain the development of neurodegenerative diseases like Alzheimer’s. The response of our model to changes in key parameters can explain the effect of aging on clearance of metabolic waste from the brain. Some studies have shown that the elastic modulus of the brain decreases with aging [65, 66], and our model predicts less fluid exchange between the SAS and the PVS when the elastic modulus is lowered (Fig. 5b). Finally, the increase of PVS width observed with aging [129] might be a reason for reduced clearance of metabolic waste from the brain (Fig. 5c). While these possibilities are admittedly speculative, they may be fertile ground for further investigation of the interaction of brain mechanics and health.

## Methods

### Modeling assumptions

Here, we present the boundary conditions and governing equations used in the simulations presented in the results section (Figs. 2, 3, 4, 5). Arteriolar wall displacements and brain tissue deformability cause the PVS to be a time-dependent domain. To properly account for the motion of the PVS, we adopted an arbitrary Lagrangian–Eulerian approach [130] (ALE). The motion of the PVS (often referred to as a mesh motion or ALE map) was modeled using a harmonic model [131] to calculate the mesh displacement ($\varvec{u}_{m}$). The ALE implementation ensures that the fluid dynamics are not affected by the choice of the model for the deformation of the fluid-filled region. The equations are presented in their more familiar Eulerian form. For a full mathematical description of the equations in the arbitrary Lagrangian–Eulerian form, please see the Additional file 1: Appendix. The simulations were performed with the assumption of axisymmetric geometry (Fig. 1).

The fluid flow in the PVS was modeled as incompressible Darcy-Brinkman flow through a highly porous region (Eqs. M1, M2, M3). The fluid velocity ($\varvec{v}_{f}$) and pressure ($p_{f}$) are the primary unknowns. The density and dynamic viscosity of the fluid are given by $\rho_{f}$ and $\mu_{f}$ respectively. The porosity and permeabilty of the PVS are given by $\zeta$ and $k_{s}$ respectively. All the parameters are described in Table 1. The stress tensor for the fluid ($\varvec{\sigma}_{f} )$ is given by eq. M3.

M1 $$\frac{{\partial \varvec{v}_{f} }}{\partial t} + \left( {\varvec{v}_{f} \cdot \nabla } \right)\varvec{v}_{f} + \frac{{\mu_{f} \zeta }}{{k_{s} \rho_{f} }}\varvec{v}_{f} - \frac{1}{{\rho_{f} }}\nabla \cdot \varvec{\sigma}_{f} = 0$$

M2 $$\nabla .\varvec{v}_{f} = 0$$

M3 $$\varvec{\sigma}_{f} = - p_{f} \varvec{I } + \mu_{f} \left( {\nabla \varvec{v}_{f} + \left( {\nabla \varvec{v}_{f} } \right)^{T} } \right)$$

For all the simulations, we used a no-slip boundary condition. This means that the fluid velocity at the arteriolar wall is given by the time derivative of the wall displacement. We assume that the arteriolar wall moves only in the radial direction. Therefore, the radial(r) and axial (z) components of fluid velocity at the arteriolar wall are given by eq. M4. The waveform of the arteriolar wall deformation for pulsations and vasodilation are shown in Fig. 2a and Fig. 4a respectively.

M4 $$\varvec{v}_{fr} = \frac{{d\varvec{u}_{wall} }}{dt} , \quad \varvec{v}_{fz} = 0$$

The brain tissue was modeled as an elastic solid. Unless otherwise stated, we modeled the brain as a compressible, De Saint–Venant-Kirchhoff material with a Poisson’s ratio ($\nu$) of 0.45. The tissue displacement ($\varvec{u}_{s}$) and velocity ($\varvec{v}_{s}$) are the primary unknowns. All the parameters are described in Table 1. The stress tensor for the solid ($\varvec{\sigma}_{s} )$, given by eq. M7, is a function of the Lagrange strain ($\varvec{\varepsilon}_{s}$) and the Lamé parameters described in eqs. M8, M9 respectively.

M5 $$\frac{{d{\varvec{u}}_{s} }}{dt} - \varvec{v}_{s} = {\mathbf{0}}$$

M6 $$\frac{{d\varvec{v}_{s} }}{dt} - \frac{{\det \varvec{F}_{s} }}{{\rho_{s} }}\nabla \cdot\varvec{\sigma}_{s} = {\mathbf{0}}$$

M7 $$\varvec{\sigma}_{s} = \frac{1}{{\det \varvec{F}_{s} }}\varvec{F}_{s} \left( {\lambda_{s} \mathrm{Tr}\left[ {\varvec{\varepsilon}_{s} } \right]\varvec{I} + 2\mu_{s} \varvec{\varepsilon}_{s} } \right)\varvec{F}_{S}^{T}$$

M8 $$\varvec{\varepsilon}_{s} = \varvec{F}_{s} \varvec{F}_{s}^{T} - \varvec{I},\quad \text{where}\quad \varvec{F}_{s} = \varvec{I} + \nabla_{\text{x}} \varvec{u}_{s}$$

M9 $$\lambda_{s} = \frac{{2v\mu_{s} }}{{\left( {1 - 2v} \right)}}$$

The interaction between the flow in the PVS and the elastic deformation of the brain was implemented using a fluid–structure interaction model. The displacement of the PVS at the Brain-PVS interface was made equal to the displacement of the brain tissue. Similarly, the velocity of the fluid at the Brain-PVS interface was made equal to the velocity of the brain tissue. The forces from the fluid flow (pressure and fluid shear) in the PVS are applied as a boundary force on the brain tissue at the Brain-PVS interface. This coupling of displacements, velocity and forces is implemented simultaneously to create a fully coupled fluid–structure interaction model [132] (eqs. M10, M11, M12).

M10 $$\varvec{u}_{m} =\varvec{u}_{s}$$

M11 $$\varvec{v}_{f} = \varvec{v}_{s}$$

M12 $$\varvec{\sigma}_{f} \cdot \varvec{n}_{f} = \varvec{\sigma}_{s} \cdot \varvec{n}_{s}$$

Here, ***u***_m_ is the mesh displacement (displacement of the fluid domain). The unit outward normals to the fluid and solid domains are represented as ***n***_f_ and ***n***_s_ respectively.

For the simulation shown in Fig. 2, the brain tissue is assumed non-compliant (fixed). The equivalent versions of eqs. M10 and M1, ***u***_m_ = 0 and ***v***_f_ = 0, are used for this case.

For simulations shown in Figs. 3 and 4, the pial opening of the PVS was connected to a flow resistance, which models fluid moving into and out of the subarachnoid space. The flow resistance was implemented as a Robin boundary condition, i.e., a flowrate-dependent pressure-like traction was applied at the pial opening of the PVS. Simulations where the subarachnoid space (SAS) was modeled as a fluid filled, porous region connected to the PVS (Additional file 7: Fig S5, Additional file 8: S8) confirmed that the Robin boundary condition [133] is adequate to simulate the flow resistance of the SAS. The other axial end of the PVS is also modelled with a Robin boundary condition representing the flow resistance of the brain parenchyma.

All our models had a Reynolds number of less than 1 for flow in the PVS (eq. M13). We also calculated the Péclet number (eq. M14) based on the diffusion coefficient (D) for Amyloid beta [134, 135]. All parameters used for the calculation of Reynolds number (Re) and Péclet number (Pe) are listed in Table 1. The fluid flow rates q_f0_, q_f50_ in eq. M15 is calculated at the top surface of the PVS (z = L_a_) and 50 µm below the brain surface respectively (z = La-50 µm).

M13 $$Re = \frac{{2\rho_{f} q_{f0} wd}}{{\mu_{f} \pi \left( {\left( {R_{1} + wd} \right)^{2} - R_{1}^{2} } \right)}}$$

M14 $$P_{e0} = \frac{{2q_{f0} wd}}{{D\pi \left( {\left( {R_{1} + wd} \right)^{2} - R_{1}^{2} } \right)}};P_{e50} = \frac{{2q_{f50} wd}}{{D\pi \left( {\left( {R_{1} + wd} \right)^{2} - R_{1}^{2} } \right)}}$$

M15 $$q_{f0} = \int_{z = la} {\varvec{v}_{f} \cdot \varvec{n}_{f} dA;} \quad q_{f50} = \int_{z = la} {\varvec{v}_{f} \cdot \varvec{n}_{f} dA}$$

### Model implementation

All the partial differential equations that govern the physics of the problem were implemented using the Galerkin finite element method [133]. All the finite element simulations were performed using COMSOL Multiphysics [136]. We used the Weak Form PDE (partial differential equation) interface in the Mathematics Module in COMSOL to implement the governing equations on an axisymmetric geometry. The strong form of the vector equations for each problem are given in the Additional file 1: Appendix. The equations are converted to a weak form in an axisymmetric (r,z) coordinate system using Wolfram Mathematica [137]. A backward difference formula (BDF) scheme was used for the time-dependent problems in COMSOL.

The particle trajectories in Fig. 4b and Additional file 10, 11, 14: Videos are estimated by calculating the fluid velocities, as observed from the mesh coordinates (which themselves change with time) in COMSOL. We then export these velocity values along with the corresponding mesh displacement values. These values are taken into MATLAB [138], where we implemented a script to calculate fluid particle trajectories using the forward-Euler time integration scheme [133].

### Surgical procedures

All procedures were performed in accordance with protocols approved by the Institutional Animal Care and Use Committee (IACUC) of Pennsylvania State University. Mice were anesthetized with isoflurane (5% induction, 2% maintenance) for all surgical procedures. The scalp was resected, and the connective tissue removed from the surface of the skull. A custom-machined titanium headbar (https://github.com/KL-Turner/Mouse-Head-Fixation) was affixed with cyanoacrylate glue (32402, Vibra-Tite) immediately posterior to the lambda cranial suture. Three self-tapping, 3/32” #000 screws (J.I. Morris) were implanted into the skull, one in each frontal bone, and one in the contralateral parietal bone. A ~ 4 mm x ~ 5 mm polished and reinforced thinned-skull window was implanted over the right hemisphere somatosensory cortex as previously described [49, 50]. After thinning, the skull was polished with 3F and 4F grit, and a #0 glass coverslip (Electrode Microscopy Sciences, #72198) was attached to the thinned portion of the skull with cyanoacrylate glue. Dental cement (Ortho-Jet) was used to seal the edges of the window and connect the headbar and headscrews. At the conclusion of the surgery the mice were returned to their home cage and allowed 2 days of recovery before being habituated to head fixation. Mice were habituated to head-fixation on a spherical treadmill (60 mm diameter) for 2–3 days before imaging. The mice were head-fixed for 30 min on the first session and the length of the session was increased to 90 min on the final session. The mice were monitored for any signs of distress during the period of habituation.

### Two-photon imaging

Prior to imaging, mice were briefly anesthetized with isoflurane and retro-orbitally injected with 50 µL of 2.5% w/v of Texas-red conjugated dextran (40 kDa; Sigma-Aldrich), then head-fixed upon a spherical treadmill. The treadmill was coated with a slip-resistant tape and connected to a rotary encoder (US Digital, E7PD-720-118) to monitor changes in velocity of the treadmill. The changes in velocity (acceleration) were used to identify periods of rest and motion. Images were collected under a Sutter moveable objective microscope with either a 16 × 0.8 NA objective or a 20 × 1.0 NA objective (Nikon). A MaiTai HP laser tuned to 920 nm was used to excite the YFP and the Texas-Red. The power exiting the objective was between 30 and 70 mW. Arteries were visually identified by their more rapid blood flow, rapid temporal dynamics of their response to locomotion, and vasomotion [51, 72, 73]. A two-channel photomultiplier setup was used to collect fluorescence from YFP and Texas-red. Images were collected at a nominal frame rate of 3–8 Hz. All the data was collected at a depth of 30–120 µm below the pial surface, and none of the arterioles imaged bifurcated before the depth at which the measurements were made.

### Data processing

A detailed flow chart of the procedure used for data processing is given Fig S11. All the data analysis was performed using MATLAB [138], except for the visual verification of displacement, which was performed using ImageJ (NIH). The code used for these displacement measurements is available on GitHub (https://github.com/kraviteja89/Thy1-displacement).

We used the red channel for motion correction (registration using discrete Fourier transform [139]) to remove movement in order to generate movies where the center of the vessel was fixed. A 3D median filter (3,3 pixels in space and 5 frames in time) was used to remove shot noise. Due to crosstalk, the images on the red channel contained some YFP fluorescence (brain tissue) in addition to the Texas Red (vessel lumen) signal. To remove this crosstalk, we used linear model to remove the YFP signal from the red channel.

M16 $$r_{i} \left( {Red Image} \right) = r_{f} \left( {Red fluoresense} \right) + \alpha \cdot g_{f} \left( {Green fluoresense} \right)$$

M17 $$g_{f} = g_{i} \left( {Green\text{Im} age} \right)$$

M18 $$\alpha = \min_{{\alpha = \left( {0,1.5} \right)}} norm\left( {r_{i} - a \cdot g_{i} } \right)$$

Here, the image in the red channel, r_i_, was assumed to be a linear combination of the actual red fluorescence, r_f_, and a weighted version,α, of the green fluorescence, g_f_ (eq. M16). The green image, g_i_, was assumed to be the actual representation of the green fluorescence (eq. M17). A constant (α) was found, that minimized the total error under an inverse model, using MATLAB’s *fminsearch* function (eq. M18). We then used eq. M16 to calculate the red fluorescence r_f_.

We then estimated the changes in arteriolar diameter using the image sequence in the red channel. The section of the image containing the arteriole was cleaned-up using the thresholding in Radon space algorithm [140]. For this method, a rectangular region containing the arteriole was manually selected. The region was transformed into Radon space for angles between 0^°^ and 180^°^ in 1^°^ increments. At each angle, the Radon transform value was rescaled between 0 and 1 to obtain a normalized value. A threshold value of 0.2 was chosen, and every value below this was set to zero. We then calculated the inverse Radon transform of the normalized values into the image space. The area of the vessel was calculated from the inverse-transformed image using the *regionprops* function in MATLAB. The velocity data (collected from the rotary encoder on the spherical treadmill) was used to create a binary vector of movement/rest during each frame [92]. The hemodynamic response function (HRF) between the binarized locomotion and the vessel diameter was calculated by fitting the parameters of a gamma distribution function [141]. Only data sets where the goodness of fit (R^2^) between the measured vessel data and the HRF-convolved function was > 0.6 were used.

The displacement of brain tissue was calculated using a piecewise rigid motion model. A reference frame was chosen by averaging 10–30 s of data when the mice were stationary (not moving). The images from the green channel were broken down into overlapping boxes of 64 × 64 pixels with 48-pixel overlap in the x or y direction. The boxes containing no appreciable fluorescence were not used in calculating displacements. This was done by looking at the peak fluorescence in each box and only using boxes that were in the top 20 percentile of peak fluorescence. For each box, the displacement was calculated using image registration [139] with the corresponding box in the reference frame. We used an iterative approach to calculate the displacements, meaning that each box was displaced by the negative value of the calculated displacement and the displacement between the reference and the corrected box was recalculated. This process was iterated five times for each box. The calculated displacement value was accepted only if the displacements converged, i.e., the displacement calculated in the last iteration was smaller than 1% of the total calculated displacement. This criterion was necessary because we observed several instances of movement of the brain tissue out of the plane of imaging. The DFT registration algorithm gives out a displacement value even when the reference and target images do not match. An example of the iterative method at work is shown in Additional file 15: Fig S11b. Additionally, we use a threshold in the error (< 70%) calculated by the DFT registration to accept or reject the calculated displacement. Above-threshold points were scrubbed from the time series and a median filter was used to fill in the scrubbed data points. A wavelet-based filter was used to denoise the resulting time series. A wavelet-based filter (“biorthogonal 3.3” wavelet in Matlab) was used because it was found to be most efficient in extracting gamma-distribution function-like signal from noise.

Only datasets of the calculated displacement time-series that met certain criteria were included. Firstly, the direction of calculated the displacement should be radially outward (± 30^°^) from the centerline of the vessel. Secondly, the displacement time-series should be well correlated with the time-series of vessel diameter changes (Pearson correlation coefficient > 0.8). This criterion was required because the brain can move in the vertical direction [142] (up to 5 µm in some cases), which resulted in noisy results while processing our planar imaging data. We found that the Pearson correlation coefficient between the vessel response and tissue response in both fluid–structure interaction and non-compliant brain paradigms using pseudo data was always greater than 0.85 for signal-to-noise ratio between 2 and 50 dB. One major drawback of this method is that we could have missed data sets where the brain tissue did not move at all. This problem can be rectified in future studies by using volumetric scans [142] instead of planar scans, and this would require data collection higher frequencies than our 3 Hz scans. Finally, we verified that the calculated displacement was visible in a projection of the image stack in time along the line of the calculated displacement. This last step was carried out in ImageJ (NIH). It is important to note that the displacements expected in both the “non-compliant brain” model and the fluid–structure interaction model meet all three criteria.

To validate our code, we tested our method on pseudo-data generated using MATLAB. We generated a random 2D array of lines oriented in different directions. We displaced the generated image uniformly radially outward with the temporal dynamics of the radial displacement given by a gamma distribution function. Varying levels of noise were added to the images to determine the robustness of the algorithm. We found that the displacements extracted by our method agree well with the input displacement, and were robust to high levels of noise (Additional file 16: Fig S12).

We used the displacements calculated from all the datasets (n = 21 vessels, 7 mice) to estimate an average peak-normalized displacement response to running (Fig. 6n). For each of the datasets, we calculated the impulse response of the radial displacement of the arteriolar wall to locomotion events using the method of deconvolution [143]. These impulse response functions were aligned so that the peak occurred at the same time and normalized to the peak value (L-infinity norm). We then calculated the impulse response functions for the radial displacement of brain tissue and applied the same time-lag as the corresponding arteriolar wall motion. We normalized the brain tissue displacements by their peak values. We plotted the average, normalized radial displacement of the arteriolar wall and the brain tissue to consider the possibility of a “non-compliant brain” model or a fluid–structure interaction model.

## Supplementary information

**Additional file 1: Appendix.** Appendix.pdf; Full mathematical formulation of the initial-boundary value problems in arbitrary Lagrangian-Eulerian coordinates.

**Additional file 2: Figure S1.** When the brain is modeled as providing a rigid boundary to the PVS, a Navier-Stokes flow predicts negligible unidirectional flow and pressure differences large enough to clearly call into question the rigidity assumption. **a.** Plot of the fluid velocity induced in the PVS by the arteriolar pulsation. Contour showing the axial velocity (velocity in the z-direction) in a cross-section of the PVS. The colors indicate the direction and magnitude of flow. Fluid velocity vectors (arrows) show a parabolic flow profile, as is expected from a Navier-Stokes model. Heartbeat pulsations drive negligible unidirectional flow with a mean flow speed(-[v_z_]) of 1.8 × 10^−3^ µm/s. To make the movements clearly visible, we scaled the displacements by a factor of 10 in post-processing. **b.** Fluid pressure in the PVS corresponding to the flow shown in **a**. Pressure changes due to fluid flow in the PVS reach several mmHg. These pressures will deform the soft brain tissue, which has a shear modulus of 1–8 kPa [51, 145] (8–60 mmHg). The dotted line shows the estimated deformation in the brain tissue (shear modulus 4kPa–Kirchhoff/De Saint-Venant elasticity with Poisson ratio of 0.45) from the pressure shown in the figure. Under these assumptions, the deformations in the brain tissue (0.08 µm) are in the same range as the peak of heartbeat driven pulsations (0.06 µm–shown in Fig 1**a**). Therefore, the deformability of brain tissue cannot be neglected even if the PVS is considered as a non-porous fluid filled channel.

**Additional file 3: Figure S2.** When the brain is modeled as providing a rigid boundary to the PVS, even when flow resistances are absent, one predicts negligible unidirectional flow with pressure differences large enough to call into question the rigidity assumption. **a.** Plot of the fluid velocity induced in the PVS by the arteriolar pulsation. Contour showing the axial velocity (velocity in the z-direction) in a cross-section of the PVS. The colors indicate the direction and magnitude of flow. Fluid velocity vectors (arrows) are provided to help the reader interpret the flow direction from the colors. Heartbeat pulsations drive negligible unidirectional flow with a mean flow speed(-[v_z_]) of 2.9 × 10^-3^ µm/s. To make the movements clearly visible, we scaled the radial displacements by a factor of 10 in post-processing. **b.** Fluid pressure in the PVS corresponding to the flow shown in **a**. Pressure changes due to fluid flow in the PVS reach several mmHg. These pressures will deform the soft brain tissue, which has a shear modulus of 1–8 kPa [51, 145] (8–60 mmHg). The dotted line shows the estimated deformation in the brain tissue (shear modulus 4kPa–Kirchhoff/De Saint-Venant elasticity with Poisson ratio of 0.45) from the pressure shown in the figure. Under these assumptions, the deformations in the brain tissue are 10 times bigger (0.71 µm) in magnitude compared the peak of heartbeat driven pulsations (0.06 µm–shown in Fig 1**a**). This shows the deformability of brain tissue cannot be neglected.

**Additional file 4: Figure S3.** Peristatic pumping can occur in models with unphysiologically long PVS. These models predict physiologically impossible pressure changes in the PVS. Note the geometry is depicted with an unequal aspect ratio in the radial (r) and axial (z) directions for viewing convenience. **a.** Plot of the fluid velocity induced in the PVS by arteriolar pulsation in the non-compliant brain model, where the length of the PVS is equal to one wavelength of the peristaltic wave (0.1 m, see Table 1). Color in the PVS shows the axial velocity (velocity in the z-direction) in a cross section of the PVS throughout the pulsation cycle. Fluid velocity vectors (arrows) are provided to help the reader interpret the flow direction from the colors. Heartbeat pulsations can drive unidirectional flow with a mean flow speed (-[v_z_]) of 143.2µm/s, but this would be accompanied by large velocity oscillations in the range of 20,000 µm/s and large pressure changes in the range of 200,000 mmHg. Note: Arteriolar and brain tissue displacements induced by arteriolar pulsations are very small (< 0.1 µm). To make the movements clearly visible, we scaled the radial displacements by 10 times in post-processing. **b.** Plot of the pressure induced in the PVS by arteriolar pulsation in the non-compliant brain model, where the length of the PVS is equal to one wavelength of the peristaltic wave (0.1m, see Table 1). No pressure is applied at both ends of the PVS. Color in the PVS shows the pressure in a cross section of the PVS throughout the pulsation cycle.

**Additional file 5: Figure S4.** Pulsation-induced fluid flows in the PVS are small in an incompressible Neo-Hookean brain model. Note the geometry is depicted with an unequal aspect ratio in the radial (r) and axial (z) directions for viewing convenience. **a.** The imposed heartbeat-driven pulsations in arteriolar radius (±0.5% of mean radius [16],R_i_) at 10 Hz, the heartrate of an un-anesthetized mouse. The pulse wave travels at 1 meter per second along the arteriolar wall, into the brain [57, 58]. **b.** Colors showing the axial velocity (velocity in the z-direction) in a cross section of the PVS, when the arteriolar wall movement is given by periodic pulsations. Fluid velocity vectors (arrows) are provided to help the reader interpret the flow direction from the colors. The white region is stationary. These plots (compare to those in Fig 3c) show that there is no significant flow into the PVS driven by arteriolar pulsations. Note: Arteriolar and brain tissue displacements induced by arteriolar pulsations are very small (< 0.1 µm). To make the movements clearly visible, we scaled the radial displacements by 10 times in post-processing. **c.** Flow out of the PVS and into the subarachnoid space, through the pial opening of the PVS. The flow rates predicted by the model with nearly incompressible (Poisson’s ratio of 0.45) (magenta) and a completely incompressible, Neo-Hookean models (blue) were nearly identical.

**Additional file 6: Figure S7.** Vasodilation-induced PVS fluid flow in a completely incompressible, Neo-Hookean model was very similar to the compressible SVK model. Note the geometry is depicted with an unequal aspect ratio in the radial (r) and axial (z) directions for viewing convenience. **a.** Plot of the prescribed arteriolar wall movement, which is identical to the one shown in Fig 4a. **b.** Plot showing the axial (z-direction) fluid velocity a cross section of the PVS, when the arteriolar wall movement is given by neural activity-driven vasodilation. A portion of the vessel lumen is shown in red to provide a sense of vasodilation. Fluid velocity vectors (arrows) are provided to help the reader interpret the flow direction from the colors. The region in white has little to no flow. These plots (very similar to the ones in Fig 4a) show that compared to heartbeat-driven pulsations (supp Fig 3b), vasodilation-driven fluid flow occurs through the entire length of the PVS and has substantially higher flow velocities. **c.** Flow out of the PVS and into the pia, through the top face of the PVS. The flow rates predicted by the model with nearly incompressible (SVK model with Poisson’s ratio of 0.45) brain tissue (magenta) and a completely incompressible, Neo-Hookean model (blue) are very similar.

**Additional file 7: Figure S5.** Pulsation-driven flows are small in simulations when the subarachnoid space (SAS) is modeled as a porous, fluid-filled region. Note the geometry is depicted with an unequal aspect ratio in the radial (r) and axial (z) directions for viewing convenience. **a.** Schematic showing the model of the penetrating arteriole used in this simulation. The brain tissue is modelled as a compliant solid. Subarachnoid space is modelled as a fluid filled region (SAS “geometry” model). **b.** Schematic showing the alternative model of the penetrating arteriole (same as Fig 3a). The Subarachnoid space is modelled as a flow resistance (R_s_) at the end of the PVS (SAS “resistance” model). The results for the SAS “resistance” model are shown in Fig 3. **c.** The imposed heartbeat-driven pulsations in arteriolar radius (±0.5% of mean radius [16],R_i_) at 10 Hz, the heartrate of an un-anesthetized mouse. The pulse wave travels at 1 meter per second along the arteriolar wall, into the brain. **d.** Plot showing the axial velocity (velocity in the z-direction) in a cross section of the PVS and the connected SAS, when the arteriolar wall movement is given by periodic pulsations. Fluid velocity vectors (arrows) are provided to help the reader interpret the flow direction from the colors. Because the fluid is incompressible, the flow speed decreases when flowing into the SAS, which has a larger area of cross section compared to the PVS. The region in white has little to no flow. These plots show that there is no significant flow into the PVS driven by arteriolar pulsations. Note: Arteriolar and brain tissue displacements induced by arteriolar pulsations are very small (< 0.1 µm). To make the movements clearly visible, we scaled the displacements by 10 times in post-processing. **e.** Plot of the fluid flow through the top face of the PVS into the SAS. The flow rates predicted by the SAS “resistance” model (magenta) and the SAS “geometry” model (blue) are very similar.

**Additional file 8: Figure S8.** Arteriolar dilations during functional hyperemia drive fluid exchange between the PVS and SAS in the SAS “geometry” model. Note the geometry is depicted with an unequal aspect ratio in the radial (r) and axial (z) directions for viewing convenience. **a.** The arteriolar wall movement is prescribed by a typical neural activity-driven vasodilation response, the same one shown in Fig 4a. **b.** Plot showing the axial velocity (velocity in the z-direction) in a cross section of the PVS and the connected SAS, when the arteriolar wall movement is given by neural activity-driven vasodilation. A portion of the vessel lumen is shown in red to provide a sense of vasodilation. Fluid velocity vectors (arrows) are provided to help the reader interpret the flow direction from the colors. Because the fluid is incompressible, the flow speed decreases when flowing into the SAS, which has a larger area of cross section compared to the PVS. The region in white has little to no flow. These plots (very similar to the ones in Fig 4a) show that compared to heartbeat-driven pulsations (supp Fig 3b), vasodilation-driven fluid flow occurs through the entire length of the PVS and has substantially higher flow velocities. Note that the scale for the radial direction is different than that in the axial direction. **c.** Flow out of the PVS and into the pia, through the top face of the PVS. The flow rates predicted by the SAS “resistance” model (magenta) and the SAS “geometry” model (blue) are almost identical.

**Additional file 9: Figure S9.** Heartbeat drives 0.5–4% (peak-to-peak) changes in arteriolar diameter. **a.** Sample image of the in-vivo fluorescence measured by two-photon microscopy following intravenous injection of FITC conjugated dextran (150 kDa) shows the cerebral vasculature near the surface of the brain (scale bar = 25 µm). Inset (scale bar = 10µm) shows a smaller region containing a segment of the arteriole, that is scanned at 30Hz to obtain arteriolar diameter changes in the typical heartrate frequencies (4-14 Hz). **b.** Sample plot of the diameter values measured for the arteriole shown in **a**. The plot shows that heartbeat drives 1.4% peak-to-peak change in diameter for this arteriole. **c.** Spectrogram shows the log power of diameter changes for the sample arteriole shown in **a**. There is a clear peak in spectral power at 5.59 Hz, which is the frequency of the heartbeat. **d.** Scatter plot shows the relation between the percentage changes in diameter (8 vessels, 6 mice) and the mean diameter at heartrate frequencies. To measure the pulsations in arterioles (diameter < 40 µm), we had to anesthetize the mice (green). The pulsations in awake animals could only be measured in large arteries (blue). Isoflurane anesthesia helped with reducing motion artifacts in measuring the small magnitude pulsations. No statistical tests were performed between the anesthetized and awake data due to the small sample size.

**Additional file** **10****: Movie SV1.** SV1_heartbeat_50s.avi; Particle tracking simulation for heartbeat driven pulsations. Duration: 50s.

**Additional file** **11****: Movie SV2.** SV2_vasodilation_50s.avi; Particle tracking simulation for functional hyperemia. Duration: 50s.

**Additional file 12: Figure S10.** The lack of negative radial displacement in the brain tissue can be attributed to the non-linear elastic response of the connective tissue in the PVS. **a.** The connective tissue in the PVS is possibly made up of extracellular matrix fibers (collagen) and fibroblasts. **b.** When arterioles dilate, the connective tissue is under compression (middle) and the fibers buckle (bend) rather than compress due to the low energy cost of bending. Therefore, there are very low elastic forces and our assumption that the forces in the PVS originate mainly from the fluid pressure is valid. **c.** When the arterioles constrict or return to their original size, the connective tissue is in tension and the fibers stretch, creating significantly larger elastic forces. In this case, our assumption that the forces in the PVS originate mainly from the fluid pressure does not hold and the fluid-structure interaction model cannot predict the behavior accurately.

**Additional file 13: Figure S6.** Deformation of the brain tissue due to the pressure changes in the PVS. Note the geometry is depicted with an unequal aspect ratio in the radial (r) and axial (z) directions for viewing convenience. a. Radial displacement contours in the brain tissue (maximum deformation, occurs at 1.16 seconds for the vasodilation profile shown in b). The brain tissue can deform by upto 1.2 µm, when the arteriole (with an initial radius of 12µm) increases its radius by 1.8 µm. b. Plot shows the change of radial displacement at the PVS-Brain interface with time. These deformations can be explained by the pressure changes in the PVS. When there is fluid outflow from the PVS, the increase in the pressure causes the brain tissue to deforms radially outward and when there is fluid influx, the brain tissue deforms radially inward. The smallest tissue displacement is at the pial surface (z = 250µm), which is the location of smallest pressure changes, as it is connected to the SAS flow resistance. The brain tissue is fixed at r = 150µm. c. Plot shows the change of radial displacement in the brain tissue at different distances from the centerline of the vessel.

**Additional file** **14****: Movie SV3.** SV3_Sample_dilation.avi; Sample imaging data showing blood vessel and brain tissue displacement. Brain tissue is marked in green (Thy1-YFP). Blood vessels are marked in magenta (Texas Red).

**Additional file 15: Figure S11.** The procedure for measuring brain tissue displacement from in-vivo imaging data collected with a two-photon laser scanning microscope. **a.** Flow chart depicting the complete procedure used to calculate displacements in the brain tissue. The procedure can be broken down into 4 major sub-sections as shown in the figure. For a full description of the procedure, see methods. **b.** A depiction of the iterative method in calculating displacements. The figure on the left shows a reference image. The intensity is shown by a Parula colormap (Matlab). The images on the right show two cases of displaced images. The one on the top is rotated by 2^°^, and can be matched to the reference image (shown in gray) by a simple displacement. After the first calculation of the displacement and correcting the displaced image, the reference and the displaced image match and further iterations of displacement calculation yield a zero value, showing that the displacement calculation has converged. The one on the bottom is rotated by 45^°^, and cannot be matched to the reference image (shown in gray) by a simple displacement. In this case, every iteration of displacement calculation yields a non-zero value and the calculation is not converged.

**Additional file 16: Figure S12.** The displacement calculation method is robust to noise. **a.** A computer generated image (512 ×  512 pixels) with randomly oriented lines. **b.** The radially-outward displacement given to the image shown in **a**. **c.** An image showing the radially-outward displacement at peak displacement (frame number 13). The initial position of the lines is shown in white and the displaced position is shown in blue. **d.** The displacement extraction procedure (shown in Figure S12) is robust to noise and predicts correct displacement. On the left, a case with low signal-to-noise ratio (0.59) is shown. The calculated displacements are very close to the actual displacement. The accuracy is comparable to the case with high signal-to-noise ratio (4.14) on the right. However, high noise results in a detection of displacement at fewer locations. The plot in the center shows that at low signal to noise ratio only 30% of the possible locations can be used for displacement calculations. Signal-to-noise ratio is calculated as the ratio of the mean signal value to the standard deviation in the noise.

## Data Availability

All the data and simulation files are available on Box (https://psu.box.com/s/xrcs2ojzs4gg0w6q2aokv5zgoybndcfw).
