# Supplementary material for: Functional hyperemia drives fluid exchange in the paravascular space
Source: Fluids Barriers CNS. 2020 Aug 20;17:52. doi: 10.1186/s12987-020-00214-3 (PMC7441569; doi:10.1186/s12987-020-00214-3)
Supplement: Supplementary file 1 — Additional file 1: Appendix. Appendix.pdf; Full mathematical formulation of the initial-boundary value problems in arbitrary Lagrangian-Eulerian coordinates. [file 12987_2020_214_MOESM1_ESM.pdf]

Appendix

A Kinematics in ALE

To describe the motion of a continuum(solid, fluid), we start with two configurations, the deformed configuration B_t, the reference configuration B_p. As shown in Fig 1, the deformed configuration B_t is the image of B_p under the map $\boldsymbol{\chi}_p$. The points in B_p, B_t are denoted by $\boldsymbol{X}_p, \boldsymbol{x}$ respectively. The displacement of material particles from the reference to the deformed configuration is given by.

$$\boldsymbol{u}(\boldsymbol{X}_p, t) := \boldsymbol{x}(\boldsymbol{X}, t) - \boldsymbol{X}_p \tag{A.1}$$

Alternatively, the Eulerian description of the displacement is given by

$$\boldsymbol{u}(\boldsymbol{x}, t) := \boldsymbol{x} - \boldsymbol{X}_p(\boldsymbol{x}, t) \tag{A.2}$$

Where $\boldsymbol{X}_p(x,t)$ is the image of $\boldsymbol{x}$ under the map $\boldsymbol{\chi}_p^{-1}$ at time t.
Denoting the material time derivative by D_t, the material particle velocity $\boldsymbol{v}$ is related to the Eulerian description of the displacement field as follows

$$\boldsymbol{v}(\boldsymbol{x}, t) := D_t\boldsymbol{u} \Rightarrow \boldsymbol{v} = \frac{\partial \boldsymbol{u}}{\partial t} + (\boldsymbol{\nabla}_x \boldsymbol{u})\boldsymbol{v} \tag{A.3}$$

where $\boldsymbol{\nabla}_x$ is the gradient with respect to $\boldsymbol{x}$ over B_t. The above equation for the material time derivative holds true for the Eulerian description of any vector quantity. It can be viewed as a property of the map $\boldsymbol{\chi}_p$

Additionally, for the ALE computation, we have the computational configuration B_c. The points in B_c are denoted by $\boldsymbol{X}_c$. As shown in Fig. A.1, the deformed configuration B_t is the image of B_c under the map χ_c. In a finite element computation, the map χ_c can be interpreted as the motion of the mesh. The displacement of the mesh gives us the relation between position of a particle in the computational domain and its position in the deformed configuration.

$$\boldsymbol{x}(\boldsymbol{X}_c, t) - \boldsymbol{X}_c =: \boldsymbol{u}_m(\boldsymbol{X}_c, t) \tag{A.4}$$

The deformation gradient and the Jacobian determinant of the mesh motion are given by

$$\boldsymbol{F}_m(\boldsymbol{X}_c, t) := \frac{\partial \boldsymbol{x}(\boldsymbol{X}_c, t)}{\partial \boldsymbol{X}_c} = \boldsymbol{I} + \boldsymbol{\nabla}_{X_c}\boldsymbol{u}_m \tag{A.5}$$

$$J_m(\boldsymbol{X}_c, t) := \det \boldsymbol{F}_m(\boldsymbol{X}_c, t) \tag{A.6}$$

where, $\boldsymbol{\nabla}_{X_c}$ is the gradient with respect to $\boldsymbol{X}_c$ over B_c.

Now, we take the material time derivative of all the quantities in eq. A.4. The material time derivative of $\boldsymbol{x}(\boldsymbol{X}_c, t)$ is the ALE representation of material particle velocity.

$$D_t\boldsymbol{x}(\boldsymbol{X}_c, t) =: \hat{\boldsymbol{v}}(\boldsymbol{X}_c, t) = \boldsymbol{v}(\boldsymbol{x}(\boldsymbol{X}_c, t), t) \tag{A.7}$$

We want to find the solution to an initial boundary value problem(or a boundary value problem) on the deforming domain. However, since our finite element computation is performed on the computational domain B_c instead of the deforming domain B_t, the primary unknown fields,for example, the stress tensor($\boldsymbol{\sigma}$) fluid

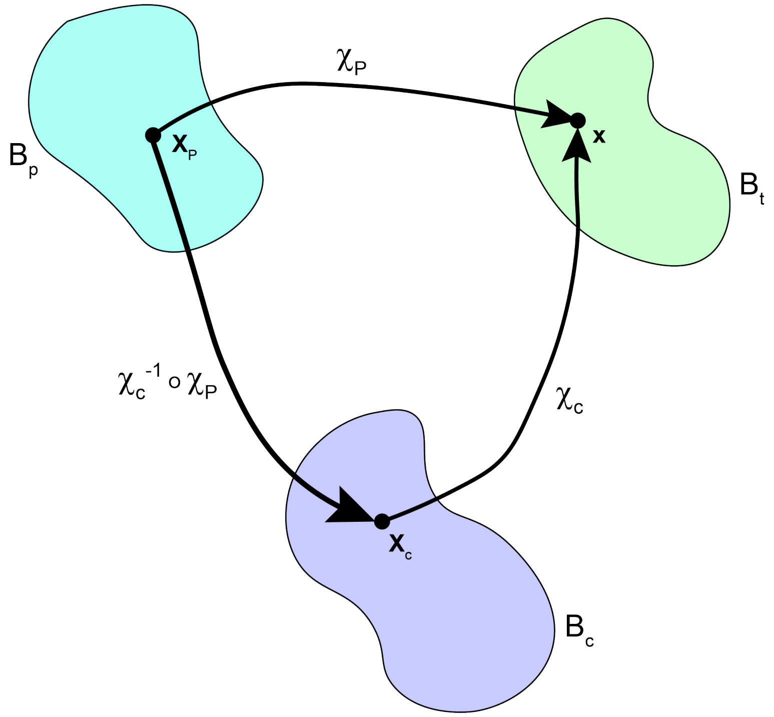

Figure A.1: Current configuration B_t is the common image of particle motion χ_p and mesh motion χ_c

velocity ($\boldsymbol{v}$) and pressure (p) will be calculated as functions of the computational coordinates $\boldsymbol{X}_c$ instead of the physical coordinates $\boldsymbol{x}$, and time t.

$$\hat{p}(\boldsymbol{X}_c, t) = p(\boldsymbol{x}, t) = p(\boldsymbol{x}(\boldsymbol{X}_c, t), t) \tag{A.8}$$

$$\hat{\boldsymbol{v}}(\boldsymbol{X}_c, t) = \boldsymbol{v}(\boldsymbol{x}, t) = \boldsymbol{v}(\boldsymbol{x}(\boldsymbol{X}_c, t), t) \tag{A.9}$$

$$\hat{\boldsymbol{\sigma}}(\boldsymbol{X}_c, t) = \boldsymbol{\sigma}(\boldsymbol{x}, t) = \boldsymbol{\sigma}(\boldsymbol{x}(\boldsymbol{X}_c, t), t) \tag{A.10}$$

We will need to express the governing equations in continuum mechanics, that contain spatial gradients and material time derivatives, in ALE coordinates. The following transformations use the deformation gradient defined in eq. A.5 and the Jacobian determinant defined in eq. A.6 to rewrite the spatial derivatives(that commonly occur in mass and momentum balance laws) in the computational domain.

For a vector $\boldsymbol{v}$:

$$\boldsymbol{\nabla}_x \boldsymbol{v} = \boldsymbol{\nabla}_{X_c} \hat{\boldsymbol{v}} \boldsymbol{F}_m^{-1} \tag{A.11}$$

$$\boldsymbol{\nabla}_x . \boldsymbol{v} = \frac{1}{J_m} Tr\left[J_m \boldsymbol{F}_m^{-1} \hat{\boldsymbol{v}}\right] \tag{A.12}$$

where, Tr is the trace operator on tensors.

For a scalar p:

$$\boldsymbol{\nabla}_x p = \boldsymbol{F}_m^{-T} \boldsymbol{\nabla}_{X_c} \hat{p} \tag{A.13}$$

For a tensor $\boldsymbol{\sigma}$

$$\boldsymbol{\nabla}_x . \boldsymbol{\sigma} = \frac{1}{J_m} \boldsymbol{\nabla}_{X_c} . \left(J_m \hat{\boldsymbol{\sigma}} \boldsymbol{F}_m^{-T}\right) \tag{A.14}$$

The material time derivative of velocity(the acceleration) estimated in the physical and computational coordinates is given as follows.

$$\boldsymbol{a}(\boldsymbol{x},t) := D_t\boldsymbol{v}(\boldsymbol{x},t) = \frac{\partial \boldsymbol{v}}{\partial t} + (\boldsymbol{\nabla}_x\boldsymbol{v})\boldsymbol{v} \tag{A.15}$$

$$\hat{\boldsymbol{a}}(\boldsymbol{X}_c,t) := D_t\hat{\boldsymbol{v}}(\boldsymbol{X}_c,t) = \frac{\partial \hat{\boldsymbol{v}}}{\partial t} + (\boldsymbol{\nabla}_{X_c}\hat{\boldsymbol{v}})\boldsymbol{F}_m^{-1}\left(\hat{\boldsymbol{v}} - \frac{\partial \boldsymbol{u}_m}{\partial t}\right) \tag{A.16}$$

The derivation of eq. A.16 is not immediately obvious. A detailed derivation is given by Donea *et al.* [3].

Further, any integral over the deformed domain can be transformed to an integral over the computational domain using the Jacobian determinant defined in eq. A.6.

$$\int_{B_t} f(\boldsymbol{x})\,\mathrm{d}\nu = \int_{B_c} J_m\hat{f}(\boldsymbol{X}_c)\,\mathrm{d}\hat{\nu} \tag{A.17}$$

where ν and $\hat{\nu}$ are infinitesimal volumes is B_t and B_c respectively.

An integral over the boundary of the deformed domain can be transformed into an integral over the boundary of the computational domain as follows

$$\int_{\partial B_t} \boldsymbol{f}(\boldsymbol{x}).\boldsymbol{n}\,\mathrm{d}a = \int_{\partial B_c} \hat{\boldsymbol{f}}(\boldsymbol{X}_c)J_m\boldsymbol{F}_m^{-T}\hat{\boldsymbol{n}}\,\mathrm{d}\hat{a} \tag{A.18}$$

where a and $\hat{a}$ are infinitesimal areas in ∂B_t and ∂B_c respectively and $\hat{\boldsymbol{n}}$ is a unit normal in the computational domain.

B Particle tracking in ALE

To track fluid particles in the computational domain(in ALE), we want to find the fluid particle coordinates ($\boldsymbol{X}_c$) as a function of time. The material time derivative of $\boldsymbol{X}_c$ is the particle velocity observed from the computational domain B_c is defined as:

$$D_t\boldsymbol{X}_c =: \dot{\boldsymbol{X}}_c \tag{B.1}$$

The material time derivative of the mesh displacement is obtained by a method similar to that in eq. A.3. In this case we use the map $\chi_c^{-1}o\chi_p$ instead of the map χ_p in eq.A.3.

$$D_t\boldsymbol{u}_m(\boldsymbol{X}_c,t) = \frac{\partial \boldsymbol{u}_m}{\partial t} + (\boldsymbol{\nabla}_{X_c}\boldsymbol{u}_m)\dot{\boldsymbol{X}}_c \tag{B.2}$$

From the finite element computation in ALE, we know the fields $\hat{\boldsymbol{v}}(\boldsymbol{X}_c,t)$ and $\boldsymbol{u}_m(\boldsymbol{X}_c,t)$. We want to find $\dot{\boldsymbol{X}}_c$ as a function of these known quantities. Using eq.s A.7, B.1 and B.2 in the material time derivative of eq. A.4 and rearranging the terms, we get

$$\hat{\boldsymbol{v}} - \dot{\boldsymbol{X}}_c = \frac{\partial \boldsymbol{u}_m}{\partial t} + (\boldsymbol{\nabla}_{X_c}\boldsymbol{u}_m)\dot{\boldsymbol{X}}_c \tag{B.3}$$

$$(\boldsymbol{I} + \boldsymbol{\nabla}_{X_c}\boldsymbol{x})\dot{\boldsymbol{X}}_c = \hat{\boldsymbol{v}} - \frac{\partial \boldsymbol{u}_m}{\partial t} \tag{B.4}$$

We can use the definition of $\boldsymbol{F}_m$ from eq. A.5 to obtain the particle velocity as seen from the computational domain

$$\dot{\boldsymbol{X}}_c = \boldsymbol{F}_m^{-1}\left(\hat{\boldsymbol{v}} - \frac{\partial \boldsymbol{u}_m}{\partial t}\right) \tag{B.5}$$

Here, we present the initial value problem to calculate fluid particle trajectories in ALE framework. This calculation is proposed as a post-processing step after the actual finite element computation. Therefore, it is

assumed that the material particle velocity $\hat{\boldsymbol{v}}(\boldsymbol{X}_c, t)$ and the mesh displacement $\boldsymbol{u}_m(\boldsymbol{X}_c, t)$ (and subsequently $\boldsymbol{F}_m$ and $\frac{\partial \boldsymbol{u}_m}{\partial t}$) are known quantities in the computational domain.

$$find\ \boldsymbol{x}(\boldsymbol{X}_c, t)\ such\ that \begin{cases} \boldsymbol{x}(\boldsymbol{X}_c, t) & = \boldsymbol{X}_c + \boldsymbol{u}_m(\boldsymbol{X}_c, t) \\ \dot{\boldsymbol{X}}_c & = \boldsymbol{F}_m^{-1}\Big(\hat{\boldsymbol{v}}(\boldsymbol{X}_c, t) - \frac{\partial \boldsymbol{u}_m(\boldsymbol{X}_c, t)}{\partial t}\Big) \\ \boldsymbol{x}(\boldsymbol{X}_c, 0) & = \boldsymbol{X}_c + \boldsymbol{u}_m(\boldsymbol{X}_c, 0) \end{cases} \tag{B.6}$$

The above initial value problem can be solved using a forward Euler integration scheme to obtain fluid particle position in the computational and deformed domains. The time integration scheme is implemented as follows to calculate the position in the computational domain at time t+dt when the position at time t is known.

$$\boldsymbol{X}_c(t + dt) = \boldsymbol{X}_c(t) + dt\boldsymbol{F}_m(\boldsymbol{X}_c(t), t)^{-1}\left(\hat{\boldsymbol{v}}(\boldsymbol{X}_c(t), t) - \frac{\partial \boldsymbol{u}_m(\boldsymbol{X}_c(t), t)}{\partial t}\right) \tag{B.7}$$

The particle position in the deformed domain is simply a consequence of the first equation of the initial value problem(eq. B.6). We can track fluid particles till the time they exit the computational domain.

There are two main reasons for choosing a forward Euler integration scheme over a backward Euler integration scheme. Firstly, the ODE described in eq. B.6 does not necessarily have a fixed point. Secondly, the values of the quantities on the right side of the ODE only exist within the computational domain, and therefore the equation is not solvable using a backward Euler scheme at the timestep when a particle leaves the computational domain.

C Initial conditions for problems

In all the intial-boundary value problems described below, the initial values and initial time-derivatives of all variables are always set to zero. This is chosen for convenience since specifying a non-zero initial condition for one variable would require the knowledge the corresponding initial values for all the other variables with coupled physics. The parameter values used in Dirichlet boundary conditions, are ramped up from zero to the specified values using step functions in Comsol multiphysics. These functions have continuous first and second derivatives with respect to time. In the subsequent sections, we will use $step1(t)$ in the equations to indicate wherever these functions are used. The results for these simulations are shown after the effect of the initial conditions are insignificant. We show the results after 20 cycles of the peristaltic wave, where the variations in the velocity and pressure fields from cycle to cycle are less than $10^{-6}\%$ of peak value.

D Darcy-Brinkman Flow in ALE coordinates

We want to solve for the fluid velocity $\boldsymbol{v}_f$ and pressure p_f in a deforming domain B_t, representing the PVS. The displacement field that defines the time-dependent deformation of the domain is denoted by $\boldsymbol{u}_m$ (same as eq. A.4). The finite element calculations are done in the computational domain B_c, where we solve for the velocity and pressure fields as a function of the computational coordinates $\boldsymbol{X}_c$. These fields $\hat{\boldsymbol{v}}_f$ and $\hat{p}_f$ are defined according to equations A.9 and A.8 respectively.

The computations are done in an axisymmetric framework. The inner and outer radius of the computational domain are R_1 & R_2 respectively, where $R_2 = R_1 + wd$. The domain has a length L_a in the z direction. See Table 1 for the list of parameters

We use a harmonic model for the mesh motion [1, 2].The governing equation for the mesh displacement, $\boldsymbol{u}_m$ on B_c is given by

$$0 = \nabla^2 \boldsymbol{u}_m \tag{D.1}$$

From here on in the document, since all the equations are presented in the computational coordinates, the X_c subscript on the spatial derivatives (gradient, divergence and laplacian) is dropped.

The boundary of B_c ($\partial B_c =: \Gamma_c$)is divided into four non-overlapping regions such that $\Gamma_c^{D1} \cup \Gamma_c^{D2} \cup \Gamma_c^{N1} \cup \Gamma_c^{N2} = \Gamma_c$, and $\Gamma_c^{D1} \cap \Gamma_c^{D2} \cap \Gamma_c^{N2} \cap \Gamma_c^{N2} = \varnothing$, where, $\varnothing$ is the empty set. The division of the boundary is shown in Fig D.1. The mesh displacement on the boundaries should reflect the motion of the physical domain. Here, the outer wall of the artery(Γ_c^{D1}) moves according to the sinusoidal wave described by eqn D.2.

$$on\ \Gamma_c^{D1}: \quad \begin{cases} u_{mr} &= b_1\ step1(t)\ \sin\left(\frac{2\pi}{\lambda}(z+ct)\right) \\ u_{mz} &= 0 \end{cases} \tag{D.2}$$

$$on\ \Gamma_c^{D2}: \quad \begin{cases} u_{mr} &= 0 \\ u_{mz} &= 0 \end{cases} \tag{D.3}$$

$$on\ \Gamma_c^{N1} \cup \Gamma_c^{N2}: \quad (\boldsymbol{\nabla}_{X_c} \boldsymbol{u}_m)\hat{\boldsymbol{n}} = \boldsymbol{0} \tag{D.4}$$

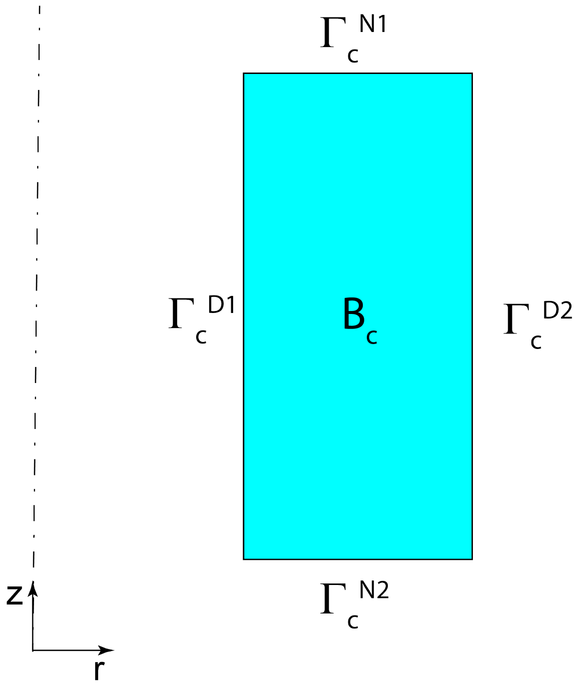

Figure D.1: Computational Domain and boundaries for the Darcy-Brinkman flow problem ALE

The governing equation for the velocity ($\hat{\boldsymbol{v}}_f$) and pressure($\hat{p}_f$) fields are given by **incompressible Darcy-Brinkman's flow**. The Eulerian form of the governing equations for incompressible Darcy-Brinkman flow are well known.

$$\boldsymbol{0} = \rho_f(\boldsymbol{a}_f - \boldsymbol{b}) - \boldsymbol{\nabla}.\boldsymbol{\sigma}_f + \frac{\zeta\mu_f}{k_s}\boldsymbol{v}_f \tag{D.5}$$

$$\boldsymbol{\sigma}_f = -\hat{p}_f\boldsymbol{I} + 2\mu_f Sym(\boldsymbol{\nabla}\boldsymbol{v}_f) \tag{D.6}$$

$$0 = \boldsymbol{\nabla}.\boldsymbol{v}_f = Tr[\boldsymbol{v}_f] \tag{D.7}$$

By converting all the derivatives to the computational coordinates(eqs A.11- A.18), we get the ALE formulation.

$$\mathbf{0} = J_m \rho_f (\hat{\boldsymbol{a}}_f - \hat{\boldsymbol{b}}) - \boldsymbol{\nabla}.\Big(J_m \hat{\boldsymbol{\sigma}}_f \boldsymbol{F}_m^{-T}\Big) + \frac{J_m \zeta \mu_f}{k_s} \hat{\boldsymbol{v}}_f \tag{D.8}$$

$$\hat{\boldsymbol{\sigma}}_f = -\hat{p}_f \boldsymbol{I} + 2\mu_f Sym\big(\boldsymbol{\nabla} \hat{\boldsymbol{v}}_f \boldsymbol{F}_m^{-1}\big) \tag{D.9}$$

$$0 = Tr\big[J_m \boldsymbol{F}_m^{-1} \hat{\boldsymbol{v}}_f\big] \tag{D.10}$$

Where $\hat{\boldsymbol{a}}_f$ is the acceleration of fluid particles defined according to eq. A.16 and $\boldsymbol{I}$ is the identity tensor. The body force, $\boldsymbol{b}$ is zero for our problem.

We use a no-slip boundary condition at the arterial wall(Γ_c^{D1}) and the brain tissue(Γ_c^{D2}) boundaries of the PVS. At the axial ends of the PVS, we assume that there is no applied traction.

$$on\ \Gamma_c^{D1} \cup \Gamma_c^{D2}: \quad \hat{\boldsymbol{v}}_f = \frac{\partial \boldsymbol{u}_m}{\partial t} \tag{D.11}$$

$$on\ \Gamma_c^{N1} \cup \Gamma_c^{N2}: \quad J_m \hat{\boldsymbol{\sigma}}_f \boldsymbol{F}_m^{-T} \hat{\boldsymbol{n}} = \mathbf{0} \tag{D.12}$$

The weak form of equations D.1, D.8 and D.10 are solved with the boundary conditions in equations D.2-D.4, D.11 and D.12. We discretize the 2D(axisymmetric) geometry using Lagrange polynomials of second order for $\boldsymbol{u}_m$ and $\hat{\boldsymbol{v}}_f$, and Lagrange polynomials of first order for $\hat{p}_f$. We use a fully coupled time dependent solver and a backward difference time integration scheme with a time step of $0.001s$.

E Fluid Structure Interaction

We have two domains B_f and B_s, representing the PVS and the Brain tissue respectively. We want to solve for the velocity($\hat{\boldsymbol{v}}_f$) and pressure($\hat{p}_f$) fields in B_f. We continue to use the hat notation for these fields because they are calculated in the computational domain that represents the un-deformed PVS. We use the Darcy-Brinkman flow model for the fluid dynamics. The displacement of the computational domain is given the field $\boldsymbol{u}_m$. In B_s, we want to calculate the solid displacement $\boldsymbol{u}_s$ and solid velocity $\boldsymbol{v}_s$. Saint-Venant-Kirchoff model is used for the solid elasticity.

For this problem, we use $\hat{\boldsymbol{n}}_f$ and $\hat{\boldsymbol{n}}_s$ to represent the outward normals to the boundaries of B_f and B_f respectively.

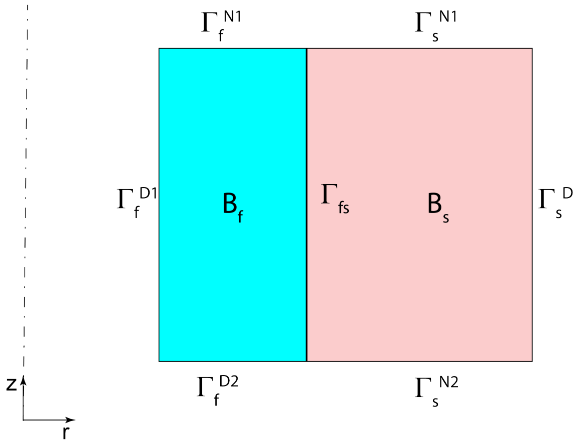

Figure E.1: Computational Domains and boundaries for the Fluid-Structure interaction problem in section 4.1.4-4.1.6

The governing equations for $u_m, \hat{\boldsymbol{v}}_f$ *and* $\hat{p}_f$ are given by equations D.1, and D.8-D.10. The boundary conditions at the fluid-structure interface (Γ_{fs}) will be discussed at the end of this section. The boundary conditions on the remaining boundaries of B_f are

$$on\Gamma_f^{D1}: \quad u_{mr} = \begin{cases} b_1 \ step1(t) \ \sin\left(\frac{2\pi}{\lambda}(z+ct)\right) & for \ pulsations \\ b(t) & for \ vasodilation \end{cases} \tag{E.1}$$

$$u_{mz} = 0 \tag{E.2}$$

$$on \ \Gamma_c^{D2} \cup \Gamma_c^{N1}: \quad (\boldsymbol{\nabla}_{X_c} \boldsymbol{u}_m)\hat{\boldsymbol{n}}_f = \boldsymbol{0} \tag{E.3}$$

The function $b(t)$ for vasodilation starts with zero initial value and is shown in main Fig 4.

$$on \ \Gamma_f^{D1}: \quad \hat{\boldsymbol{v}_f} = \frac{\partial \boldsymbol{u}_m}{\partial t} \tag{E.4}$$

$$on \ \Gamma_f^{D2}: \quad \hat{v_{fz}} = 0 \tag{E.5}$$

$$on \ \Gamma_f^{N1}: \quad J_m \hat{\boldsymbol{\sigma}}_f \boldsymbol{F}_m^{-T} \hat{\boldsymbol{n}}_f = -R_s Q_1 \hat{\boldsymbol{n}}_f \tag{E.6}$$

$$where, \ Q_1 = \zeta_f \hat{\boldsymbol{v}}_f . \left(J_m \boldsymbol{F}_m^{-T} \hat{\boldsymbol{n}}_f\right) \tag{E.7}$$

Here, Q_1 is the flow rate out of the PVS through the face Γ_f^{N1}. R_s is the resistance of the subarachnoid space, which is taken to be 1% the flow resistance of the PVS. Equation E.6 is a Robin boundary condition, where the traction at a surface is proportional to the flow rate out of the surface. It serves as a lumped model for the subarachnoid space.

For the domain B_s, the mesh motion is given by the solid displacement $\boldsymbol{u}_s$. This is the Lagrangian framework, used commonly in solid mechanics. The deformation gradient $\boldsymbol{F}_s$ and the Jacobian determinant J_s are defined similar to those in eqs A.5 and A.6. The strain energy for the Saint-venant-Kirchoff elastic model and the first Piola-Kirchoff stress are given by

$$W_s = \frac{\lambda_s}{2}(Tr[\boldsymbol{\mathcal{E}}_s])^2 + \mu_s Tr[\boldsymbol{\mathcal{E}}_s^2] \tag{E.8}$$

$$\boldsymbol{P}_s = \frac{\partial W_s}{\partial \boldsymbol{F}_s} \tag{E.9}$$

$$\boldsymbol{\mathcal{E}}_s := \frac{1}{2}\left(\boldsymbol{F}_s^T \boldsymbol{F}_s - \boldsymbol{I}\right) \tag{E.10}$$

here, Tr is the trace operator in tensors.

Since the mesh in the domain B_s follows the material particles, the material time derivatives in B_s are equal to the partial derivatives. The governing equations for $\boldsymbol{u}_s, \boldsymbol{v}_s$ are given by,

$$\boldsymbol{v}_s = \frac{\partial \boldsymbol{u}_s}{\partial t} \tag{E.11}$$

$$\boldsymbol{0} = \rho_s\left(\frac{\partial \boldsymbol{v}_s}{\partial t} - \boldsymbol{b}_s\right) - \boldsymbol{\nabla P}_s \tag{E.12}$$

For our problem, the body forces b_s are zero in B_s.

The boundary conditions on $\Gamma_s - \Gamma_{fs}$ are as follows.

$$on\ \Gamma_s^D: \quad \begin{cases} u_{sr} = 0 \\ v_{sr} = 0 \end{cases} \tag{E.13}$$

$$on\ \Gamma_s^D \cap \Gamma_s^{N2} \quad \begin{cases} u_{sz} = 0 \\ v_{sz} = 0 \end{cases} \tag{E.14}$$

$$on\ \Gamma_s^{N1} \cup \Gamma_s^{N2}: \quad \boldsymbol{P}_s \hat{\boldsymbol{n}}_s = \boldsymbol{0} \tag{E.15}$$

At the solid fluid interface, we enforce continuity of velocity and traction.

$$on\ \Gamma_{fs}: \quad \hat{\boldsymbol{v}}_f = \boldsymbol{v}_s \tag{E.16}$$

$$on\ \Gamma_{fs}: \quad \boldsymbol{0} = J_m \hat{\boldsymbol{\sigma}}_f \boldsymbol{F}_m^{-T} \hat{\boldsymbol{n}}_f + \boldsymbol{P}_s \hat{\boldsymbol{n}}_s \tag{E.17}$$

The weak form of equations D.1, D.8, D.10 on B_f and E.11 and E.12 on B_s are solved with the boundary conditions in equations E.1-E.7 and E.13-E.17. We discretize the 2D(axisymmetric) geometry using Lagrange polynomials of second order for $\boldsymbol{u}_m, \hat{\boldsymbol{v}}_f, \boldsymbol{u}_s\ and\ \boldsymbol{v}_s$ and Lagrange polynomials of first order for $\hat{p}_f, p_s, q_s\ and\ j_s$. We use a fully coupled time dependent solver and a backward difference time integration scheme with a time step of $0.0001s$ for arterial pulsations and $0.0001s$ for functional hyperemia.

F Volume exchange fraction

We are interested in calculating the maximum fraction of the fluid in the PVS exchanged with the SAS. To do this, we calculate the change of the fluid volume in the PVS at every time step, divide it by the initial volume of fluid in the PVS, and find the maximum value of this ratio.

Initial volume of fluid in the PVS can be calculated in the undeformed configuration of the fluid domain B_f. For an fluid volume fraction(porosity) of ζ, we have the initial volume of fluid V_i

$$V_i = \int_{B_f} \zeta \mathrm{d}\hat{v} \tag{F.1}$$

Since only the fluid leaves the PVS, the change in the fluid volume of the PVS is equal to the change in the total volume of the PVS. The calculation of the initial and deformed volume of the PVS is straight forward from A.17. The change in the PVS volume, δV_t at any time t can be calculated over the undeformed configuration of the fluid domain B_f.

$$\delta V_t = \int_{B_f} (1 - J_m(\boldsymbol{X}_c, t)) \mathrm{d}\hat{v} \tag{F.2}$$

From these two quantities, we can calculate the fraction of PVS fluid volume exchanged with the SAS at any time t. The volume exchange fraction Q_f is the maximum value of this quantity over the entire time period of the simulation ($0 < t < T$).

$$Q_f = \max_{0<t<T} \frac{\delta V_t}{V_i} \tag{F.3}$$

References

[1] T. Dunne and R. Rannacher. Adaptive finite element approximation of fluid-structure interaction based on an eulerian variational formulation. In *Fluid-structure interaction*, pages 110–145. Springer, 2006.

[2] T. Richter and T. Wick. Finite elements for fluid–structure interaction in ale and fully eulerian coordinates. *Computer Methods in Applied Mechanics and Engineering*, 199(41-44):2633–2642, 2010.

[3] J. Sarrate, A. Huerta, and J. Donea. Arbitrary lagrangian–eulerian formulation for fluid–rigid body interaction. *Computer Methods in Applied Mechanics and Engineering*, 190(24-25):3171–3188, 2001.
