# Supplementary figures and images for: Functional hyperemia drives fluid exchange in the paravascular space
Source: Fluids Barriers CNS. 2020 Aug 20;17:52. doi: 10.1186/s12987-020-00214-3 (PMC7441569; doi:10.1186/s12987-020-00214-3)

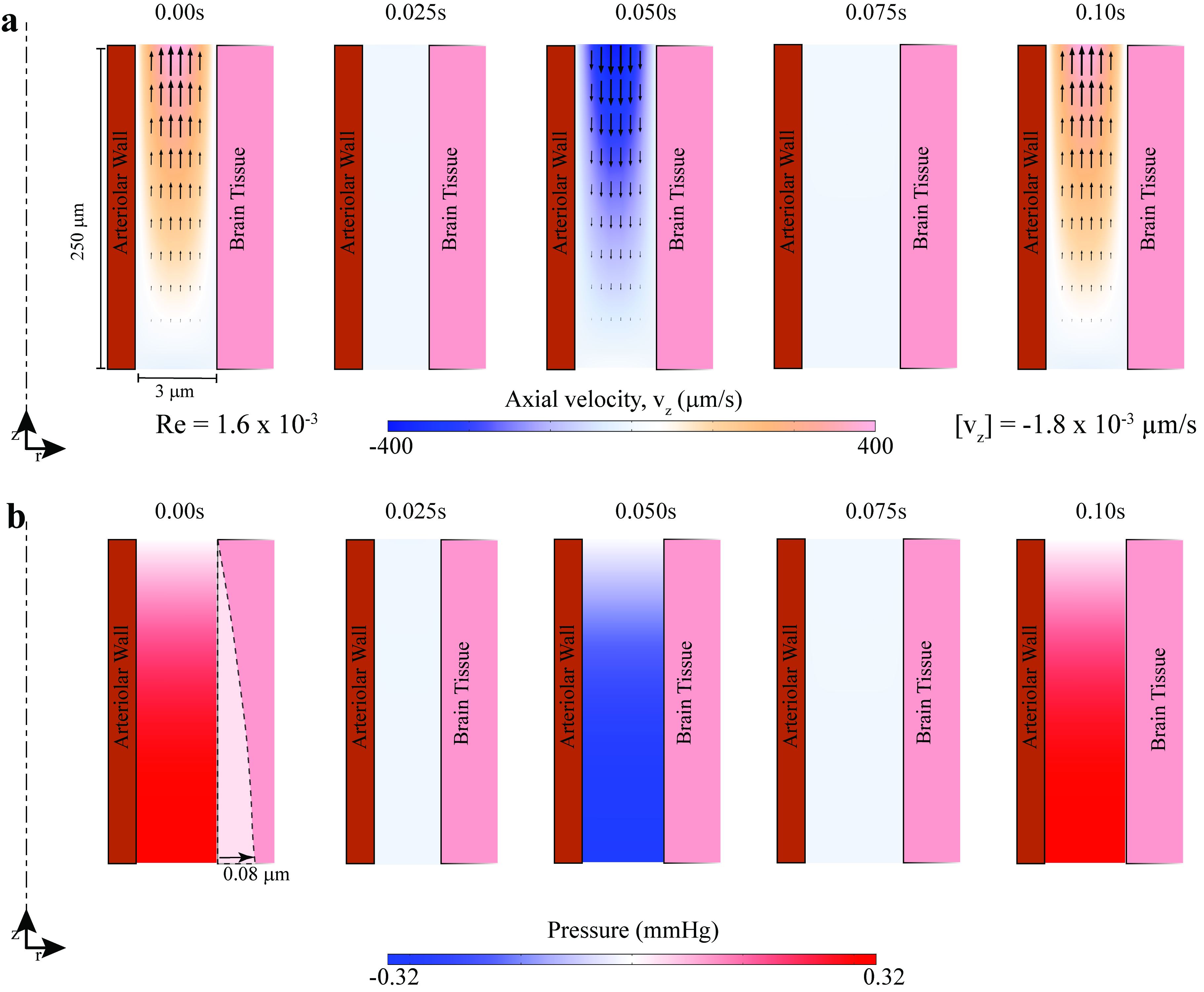

Supplement: Supplementary file 2 — Additional file 2: Figure S1. When the brain is modeled as providing a rigid boundary to the PVS, a Navier-Stokes flow predicts negligible unidirectional flow and pressure differences large enough to clearly call into question the rigidity assumption. a. Plot of the fluid velocity induced in the PVS by the arteriolar pulsation. Contour showing the axial velocity (velocity in the z-direction) in a cross-section of the PVS. The colors indicate the direction and magnitude of flow. Fluid velocity vectors (arrows) show a parabolic flow profile, as is expected from a Navier-Stokes model. Heartbeat pulsations drive negligible unidirectional flow with a mean flow speed(-[vz]) of 1.8 × 10−3 µm/s. To make the movements clearly visible, we scaled the displacements by a factor of 10 in post-processing. b. Fluid pressure in the PVS corresponding to the flow shown in a. Pressure changes due to fluid flow in the PVS reach several mmHg. These pressures will deform the soft brain tissue, which has a shear modulus of 1–8 kPa [51, 145] (8–60 mmHg). The dotted line shows the estimated deformation in the brain tissue (shear modulus 4kPa–Kirchhoff/De Saint-Venant elasticity with Poisson ratio of 0.45) from the pressure shown in the figure. Under these assumptions, the deformations in the brain tissue (0.08 µm) are in the same range as the peak of heartbeat driven pulsations (0.06 µm–shown in Fig 1a). Therefore, the deformability of brain tissue cannot be neglected even if the PVS is considered as a non-porous fluid filled channel. [file 12987_2020_214_MOESM2_ESM.tif]

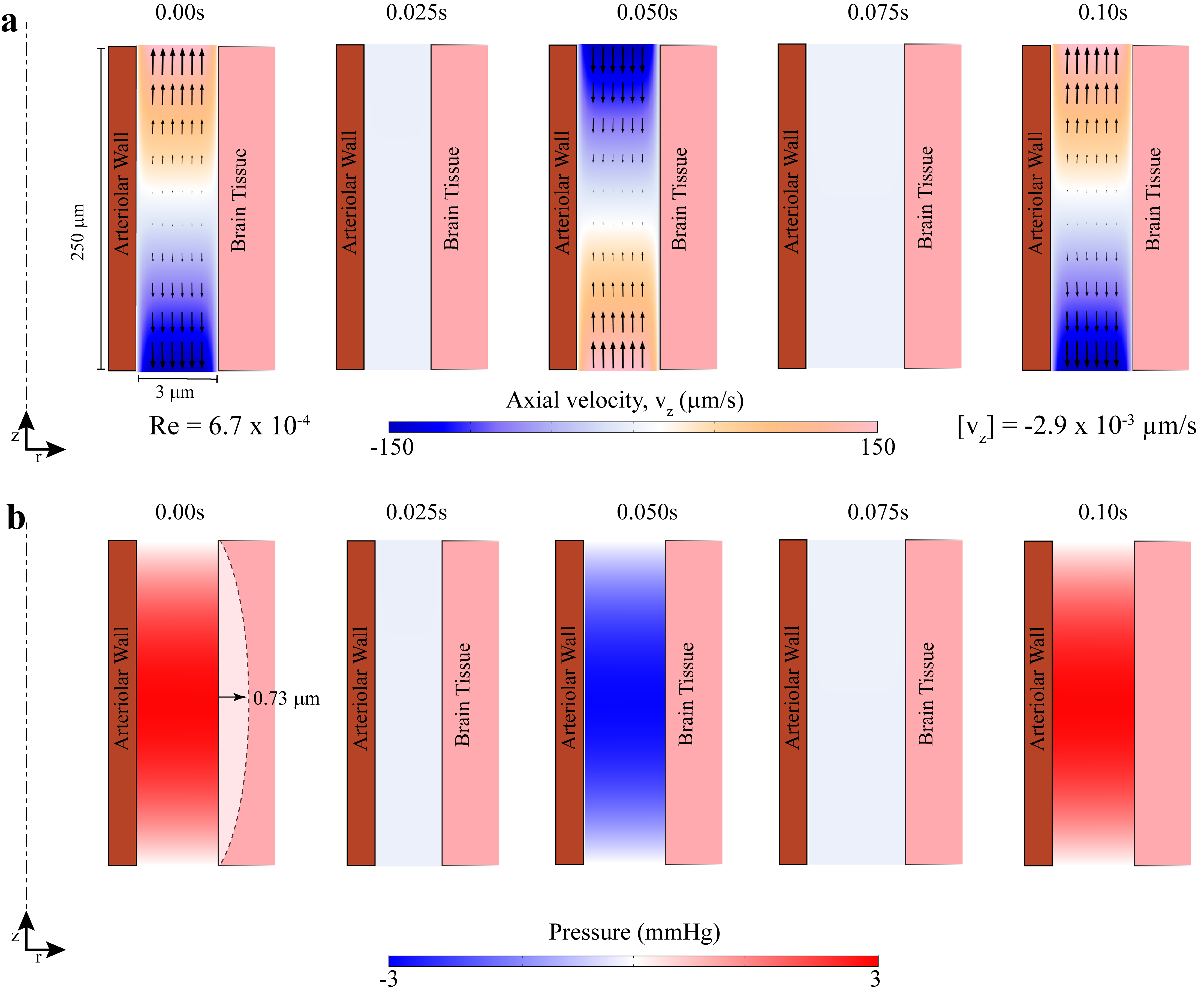

Supplement: Supplementary file 3 — Additional file 3: Figure S2. When the brain is modeled as providing a rigid boundary to the PVS, even when flow resistances are absent, one predicts negligible unidirectional flow with pressure differences large enough to call into question the rigidity assumption. a. Plot of the fluid velocity induced in the PVS by the arteriolar pulsation. Contour showing the axial velocity (velocity in the z-direction) in a cross-section of the PVS. The colors indicate the direction and magnitude of flow. Fluid velocity vectors (arrows) are provided to help the reader interpret the flow direction from the colors. Heartbeat pulsations drive negligible unidirectional flow with a mean flow speed(-[vz]) of 2.9 × 10-3 µm/s. To make the movements clearly visible, we scaled the radial displacements by a factor of 10 in post-processing. b. Fluid pressure in the PVS corresponding to the flow shown in a. Pressure changes due to fluid flow in the PVS reach several mmHg. These pressures will deform the soft brain tissue, which has a shear modulus of 1–8 kPa [51, 145] (8–60 mmHg). The dotted line shows the estimated deformation in the brain tissue (shear modulus 4kPa–Kirchhoff/De Saint-Venant elasticity with Poisson ratio of 0.45) from the pressure shown in the figure. Under these assumptions, the deformations in the brain tissue are 10 times bigger (0.71 µm) in magnitude compared the peak of heartbeat driven pulsations (0.06 µm–shown in Fig 1a). This shows the deformability of brain tissue cannot be neglected. [file 12987_2020_214_MOESM3_ESM.tif]

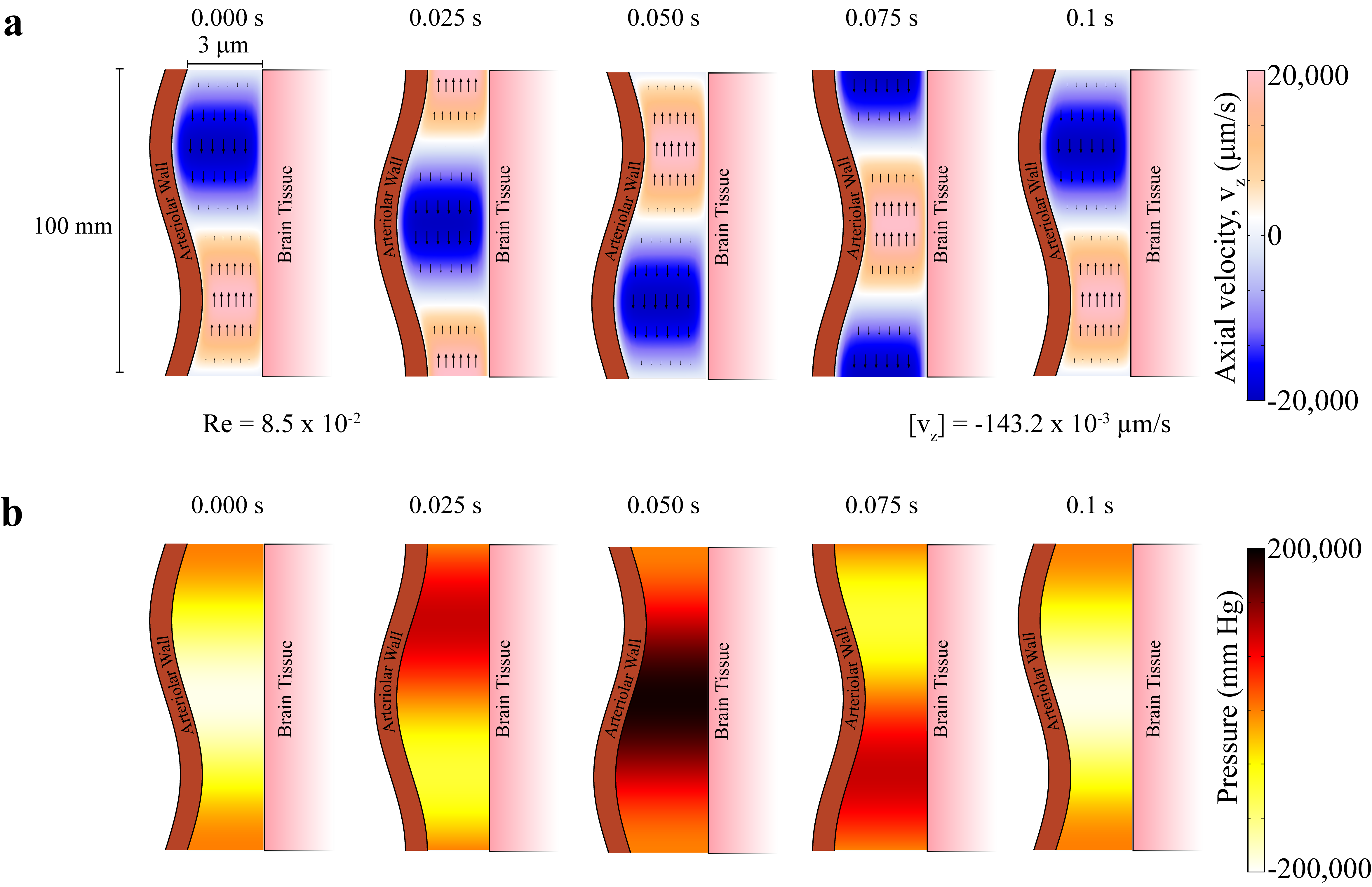

Supplement: Supplementary file 4 — Additional file 4: Figure S3. Peristatic pumping can occur in models with unphysiologically long PVS. These models predict physiologically impossible pressure changes in the PVS. Note the geometry is depicted with an unequal aspect ratio in the radial (r) and axial (z) directions for viewing convenience. a. Plot of the fluid velocity induced in the PVS by arteriolar pulsation in the non-compliant brain model, where the length of the PVS is equal to one wavelength of the peristaltic wave (0.1 m, see Table 1). Color in the PVS shows the axial velocity (velocity in the z-direction) in a cross section of the PVS throughout the pulsation cycle. Fluid velocity vectors (arrows) are provided to help the reader interpret the flow direction from the colors. Heartbeat pulsations can drive unidirectional flow with a mean flow speed (-[vz]) of 143.2µm/s, but this would be accompanied by large velocity oscillations in the range of 20,000 µm/s and large pressure changes in the range of 200,000 mmHg. Note: Arteriolar and brain tissue displacements induced by arteriolar pulsations are very small (< 0.1 µm). To make the movements clearly visible, we scaled the radial displacements by 10 times in post-processing. b. Plot of the pressure induced in the PVS by arteriolar pulsation in the non-compliant brain model, where the length of the PVS is equal to one wavelength of the peristaltic wave (0.1m, see Table 1). No pressure is applied at both ends of the PVS. Color in the PVS shows the pressure in a cross section of the PVS throughout the pulsation cycle. [file 12987_2020_214_MOESM4_ESM.tif]

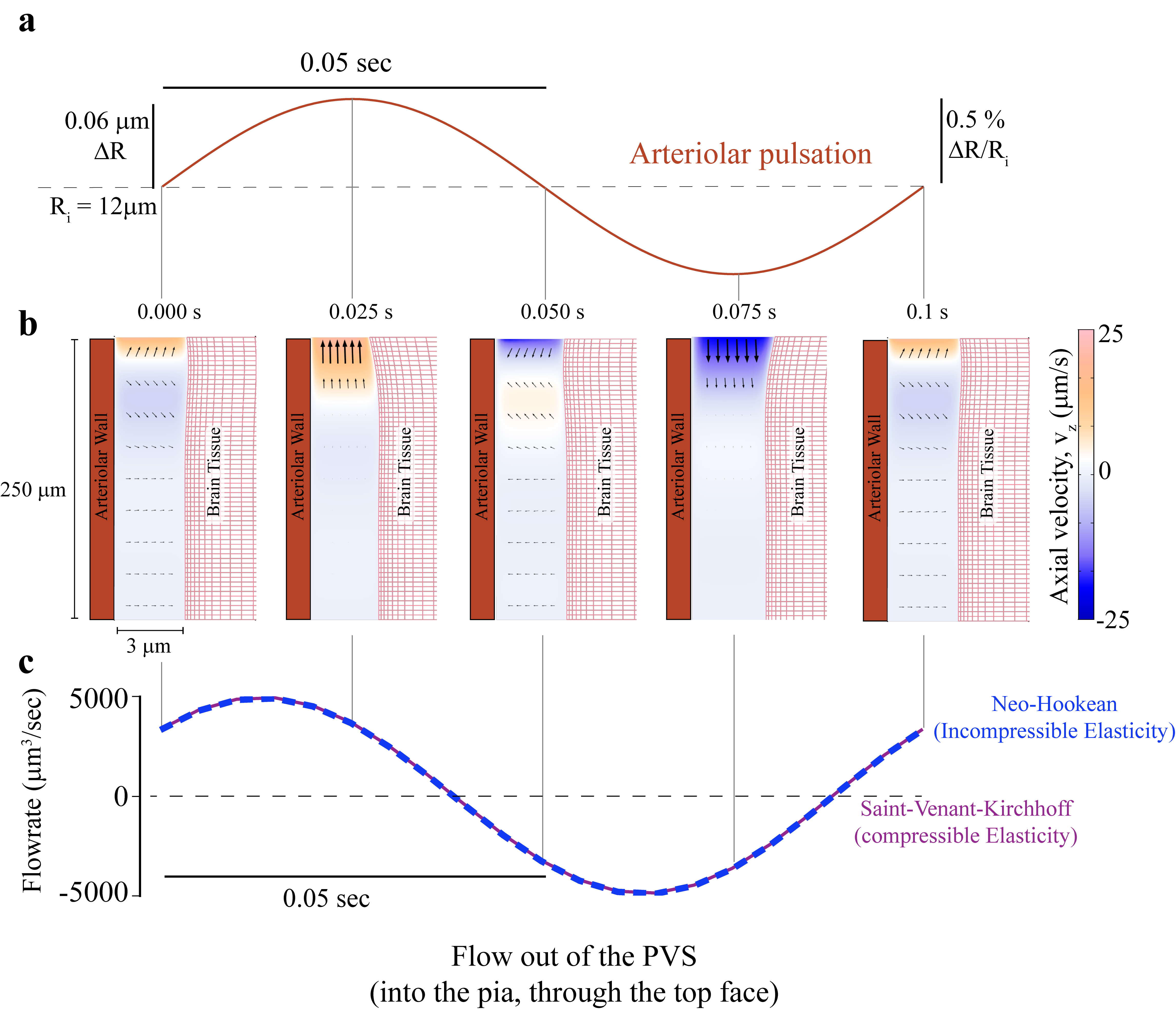

Supplement: Supplementary file 5 — Additional file 5: Figure S4. Pulsation-induced fluid flows in the PVS are small in an incompressible Neo-Hookean brain model. Note the geometry is depicted with an unequal aspect ratio in the radial (r) and axial (z) directions for viewing convenience. a. The imposed heartbeat-driven pulsations in arteriolar radius (±0.5% of mean radius [16],Ri) at 10 Hz, the heartrate of an un-anesthetized mouse. The pulse wave travels at 1 meter per second along the arteriolar wall, into the brain [57, 58]. b. Colors showing the axial velocity (velocity in the z-direction) in a cross section of the PVS, when the arteriolar wall movement is given by periodic pulsations. Fluid velocity vectors (arrows) are provided to help the reader interpret the flow direction from the colors. The white region is stationary. These plots (compare to those in Fig 3c) show that there is no significant flow into the PVS driven by arteriolar pulsations. Note: Arteriolar and brain tissue displacements induced by arteriolar pulsations are very small (< 0.1 µm). To make the movements clearly visible, we scaled the radial displacements by 10 times in post-processing. c. Flow out of the PVS and into the subarachnoid space, through the pial opening of the PVS. The flow rates predicted by the model with nearly incompressible (Poisson’s ratio of 0.45) (magenta) and a completely incompressible, Neo-Hookean models (blue) were nearly identical. [file 12987_2020_214_MOESM5_ESM.tif]

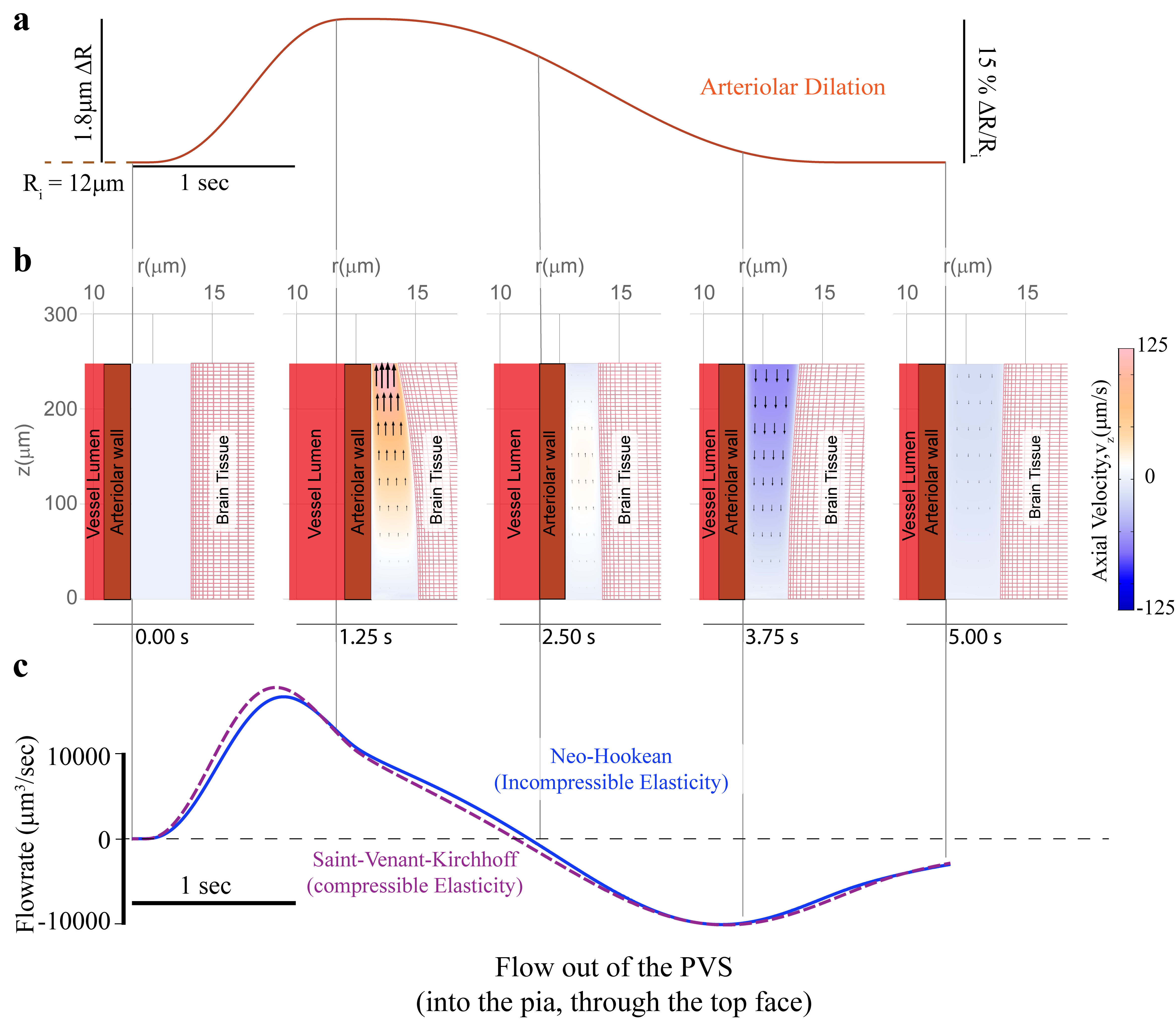

Supplement: Supplementary file 6 — Additional file 6: Figure S7. Vasodilation-induced PVS fluid flow in a completely incompressible, Neo-Hookean model was very similar to the compressible SVK model. Note the geometry is depicted with an unequal aspect ratio in the radial (r) and axial (z) directions for viewing convenience. a. Plot of the prescribed arteriolar wall movement, which is identical to the one shown in Fig 4a. b. Plot showing the axial (z-direction) fluid velocity a cross section of the PVS, when the arteriolar wall movement is given by neural activity-driven vasodilation. A portion of the vessel lumen is shown in red to provide a sense of vasodilation. Fluid velocity vectors (arrows) are provided to help the reader interpret the flow direction from the colors. The region in white has little to no flow. These plots (very similar to the ones in Fig 4a) show that compared to heartbeat-driven pulsations (supp Fig 3b), vasodilation-driven fluid flow occurs through the entire length of the PVS and has substantially higher flow velocities. c. Flow out of the PVS and into the pia, through the top face of the PVS. The flow rates predicted by the model with nearly incompressible (SVK model with Poisson’s ratio of 0.45) brain tissue (magenta) and a completely incompressible, Neo-Hookean model (blue) are very similar. [file 12987_2020_214_MOESM6_ESM.tif]

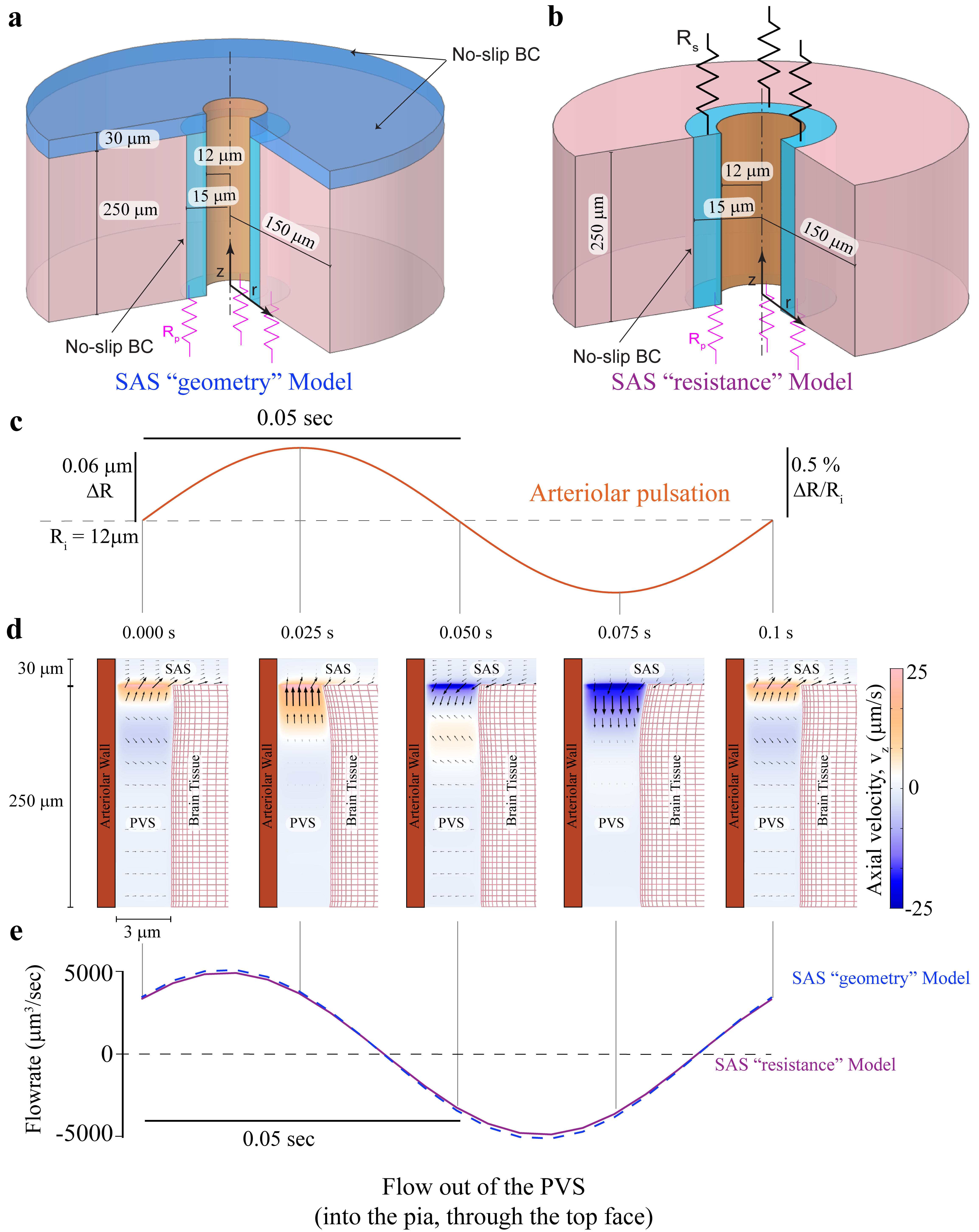

Supplement: Supplementary file 7 — Additional file 7: Figure S5. Pulsation-driven flows are small in simulations when the subarachnoid space (SAS) is modeled as a porous, fluid-filled region. Note the geometry is depicted with an unequal aspect ratio in the radial (r) and axial (z) directions for viewing convenience. a. Schematic showing the model of the penetrating arteriole used in this simulation. The brain tissue is modelled as a compliant solid. Subarachnoid space is modelled as a fluid filled region (SAS “geometry” model). b. Schematic showing the alternative model of the penetrating arteriole (same as Fig 3a). The Subarachnoid space is modelled as a flow resistance (Rs) at the end of the PVS (SAS “resistance” model). The results for the SAS “resistance” model are shown in Fig 3. c. The imposed heartbeat-driven pulsations in arteriolar radius (±0.5% of mean radius [16],Ri) at 10 Hz, the heartrate of an un-anesthetized mouse. The pulse wave travels at 1 meter per second along the arteriolar wall, into the brain. d. Plot showing the axial velocity (velocity in the z-direction) in a cross section of the PVS and the connected SAS, when the arteriolar wall movement is given by periodic pulsations. Fluid velocity vectors (arrows) are provided to help the reader interpret the flow direction from the colors. Because the fluid is incompressible, the flow speed decreases when flowing into the SAS, which has a larger area of cross section compared to the PVS. The region in white has little to no flow. These plots show that there is no significant flow into the PVS driven by arteriolar pulsations. Note: Arteriolar and brain tissue displacements induced by arteriolar pulsations are very small (< 0.1 µm). To make the movements clearly visible, we scaled the displacements by 10 times in post-processing. e. Plot of the fluid flow through the top face of the PVS into the SAS. The flow rates predicted by the SAS “resistance” model (magenta) and the SAS “geometry” model (blue) are very similar. [file 12987_2020_214_MOESM7_ESM.tif]

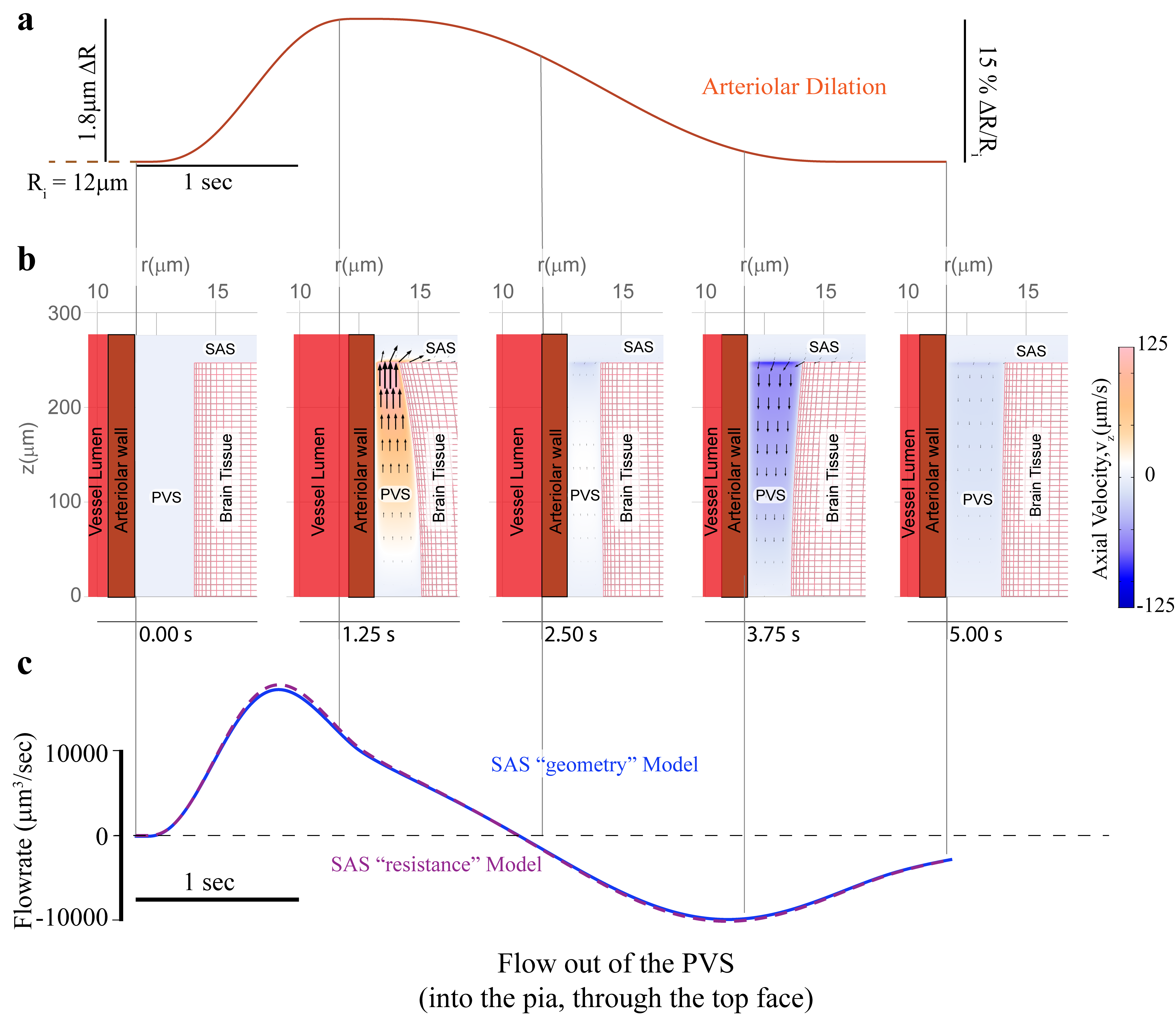

Supplement: Supplementary file 8 — Additional file 8: Figure S8. Arteriolar dilations during functional hyperemia drive fluid exchange between the PVS and SAS in the SAS “geometry” model. Note the geometry is depicted with an unequal aspect ratio in the radial (r) and axial (z) directions for viewing convenience. a. The arteriolar wall movement is prescribed by a typical neural activity-driven vasodilation response, the same one shown in Fig 4a. b. Plot showing the axial velocity (velocity in the z-direction) in a cross section of the PVS and the connected SAS, when the arteriolar wall movement is given by neural activity-driven vasodilation. A portion of the vessel lumen is shown in red to provide a sense of vasodilation. Fluid velocity vectors (arrows) are provided to help the reader interpret the flow direction from the colors. Because the fluid is incompressible, the flow speed decreases when flowing into the SAS, which has a larger area of cross section compared to the PVS. The region in white has little to no flow. These plots (very similar to the ones in Fig 4a) show that compared to heartbeat-driven pulsations (supp Fig 3b), vasodilation-driven fluid flow occurs through the entire length of the PVS and has substantially higher flow velocities. Note that the scale for the radial direction is different than that in the axial direction. c. Flow out of the PVS and into the pia, through the top face of the PVS. The flow rates predicted by the SAS “resistance” model (magenta) and the SAS “geometry” model (blue) are almost identical. [file 12987_2020_214_MOESM8_ESM.tif]

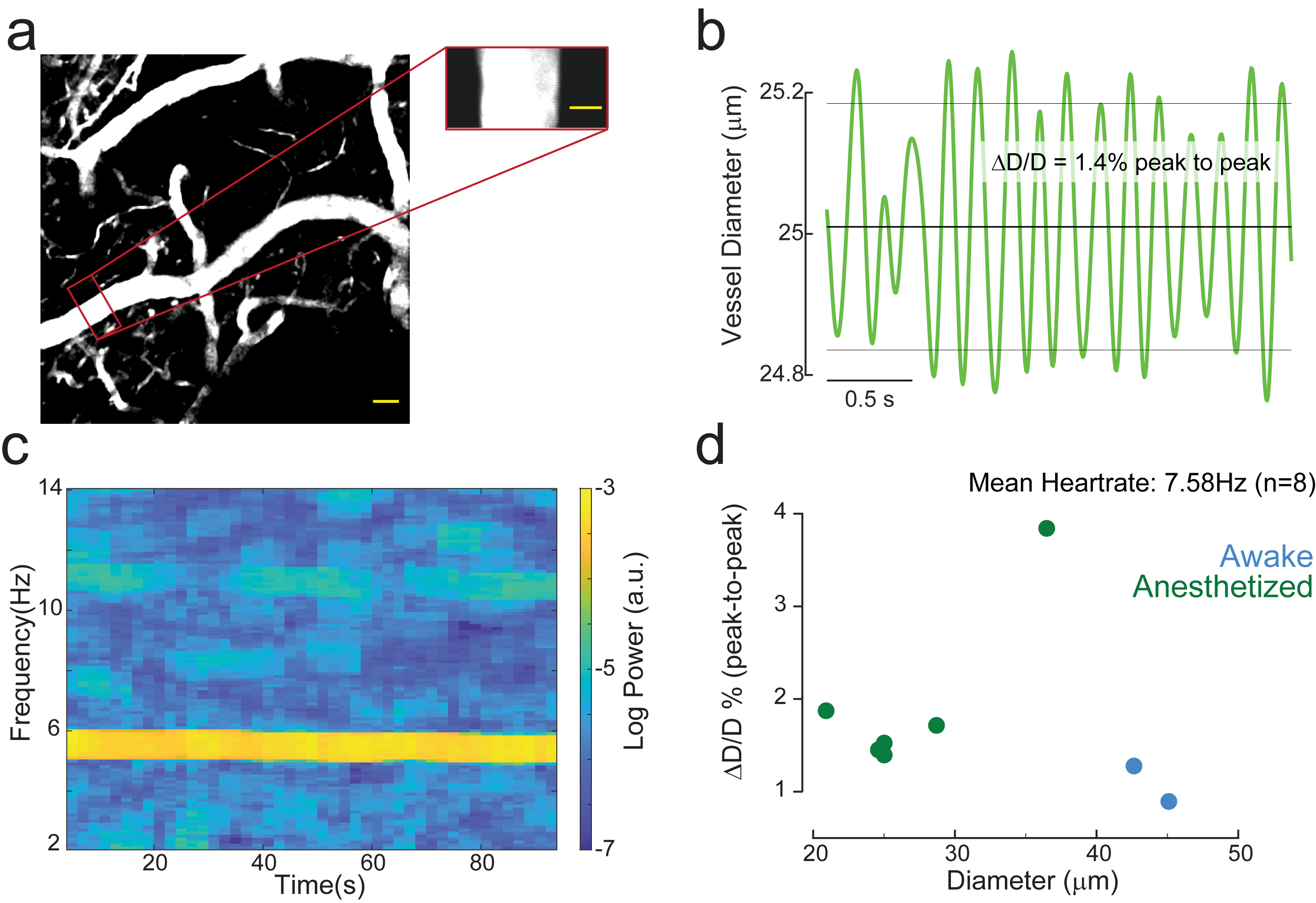

Supplement: Supplementary file 9 — Additional file 9: Figure S9. Heartbeat drives 0.5–4% (peak-to-peak) changes in arteriolar diameter. a. Sample image of the in-vivo fluorescence measured by two-photon microscopy following intravenous injection of FITC conjugated dextran (150 kDa) shows the cerebral vasculature near the surface of the brain (scale bar = 25 µm). Inset (scale bar = 10µm) shows a smaller region containing a segment of the arteriole, that is scanned at 30Hz to obtain arteriolar diameter changes in the typical heartrate frequencies (4-14 Hz). b. Sample plot of the diameter values measured for the arteriole shown in a. The plot shows that heartbeat drives 1.4% peak-to-peak change in diameter for this arteriole. c. Spectrogram shows the log power of diameter changes for the sample arteriole shown in a. There is a clear peak in spectral power at 5.59 Hz, which is the frequency of the heartbeat. d. Scatter plot shows the relation between the percentage changes in diameter (8 vessels, 6 mice) and the mean diameter at heartrate frequencies. To measure the pulsations in arterioles (diameter < 40 µm), we had to anesthetize the mice (green). The pulsations in awake animals could only be measured in large arteries (blue). Isoflurane anesthesia helped with reducing motion artifacts in measuring the small magnitude pulsations. No statistical tests were performed between the anesthetized and awake data due to the small sample size. [file 12987_2020_214_MOESM9_ESM.tif]

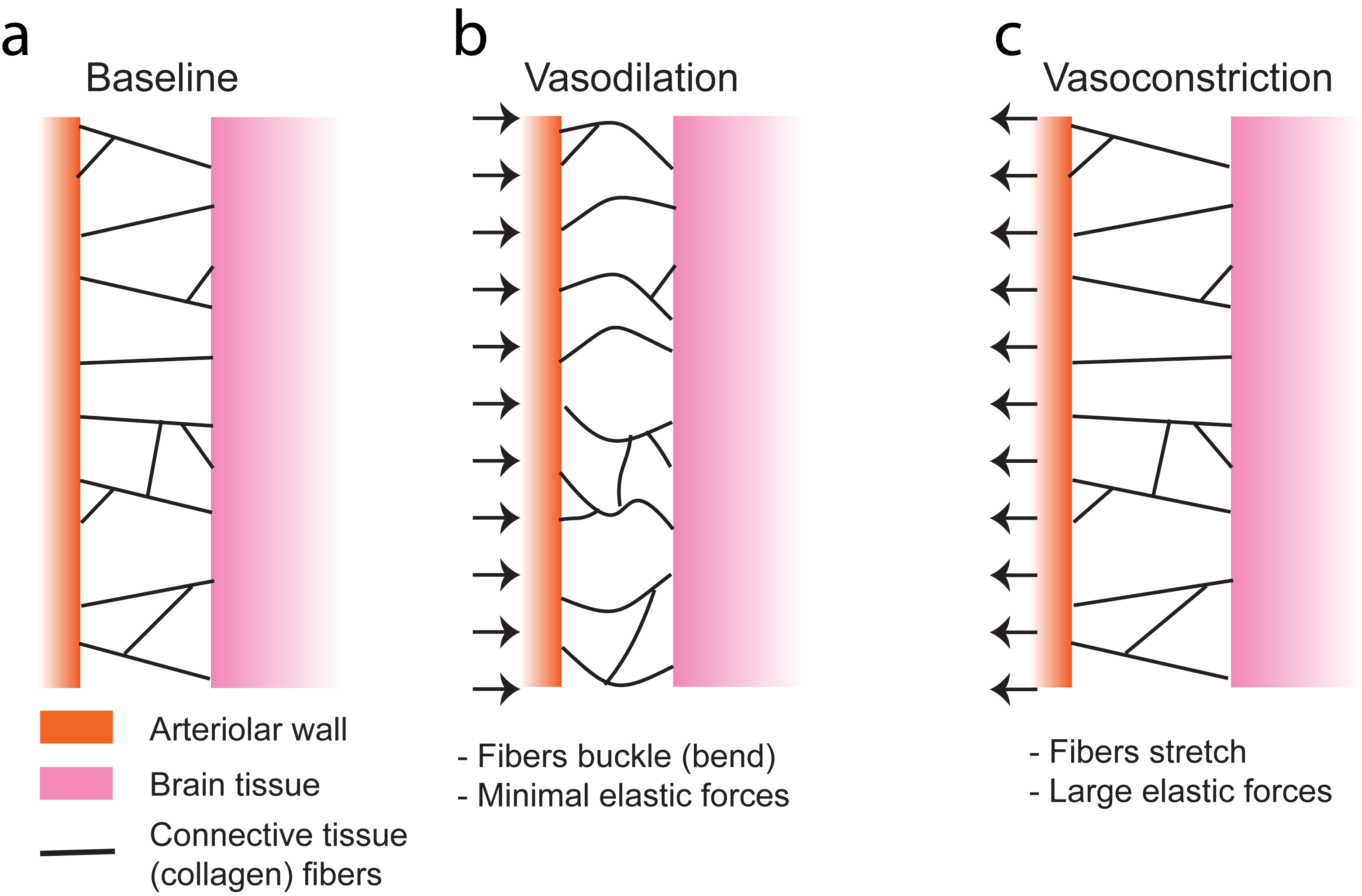

Supplement: Supplementary file 12 — Additional file 12: Figure S10. The lack of negative radial displacement in the brain tissue can be attributed to the non-linear elastic response of the connective tissue in the PVS. a. The connective tissue in the PVS is possibly made up of extracellular matrix fibers (collagen) and fibroblasts. b. When arterioles dilate, the connective tissue is under compression (middle) and the fibers buckle (bend) rather than compress due to the low energy cost of bending. Therefore, there are very low elastic forces and our assumption that the forces in the PVS originate mainly from the fluid pressure is valid. c. When the arterioles constrict or return to their original size, the connective tissue is in tension and the fibers stretch, creating significantly larger elastic forces. In this case, our assumption that the forces in the PVS originate mainly from the fluid pressure does not hold and the fluid-structure interaction model cannot predict the behavior accurately. [file 12987_2020_214_MOESM12_ESM.tif]

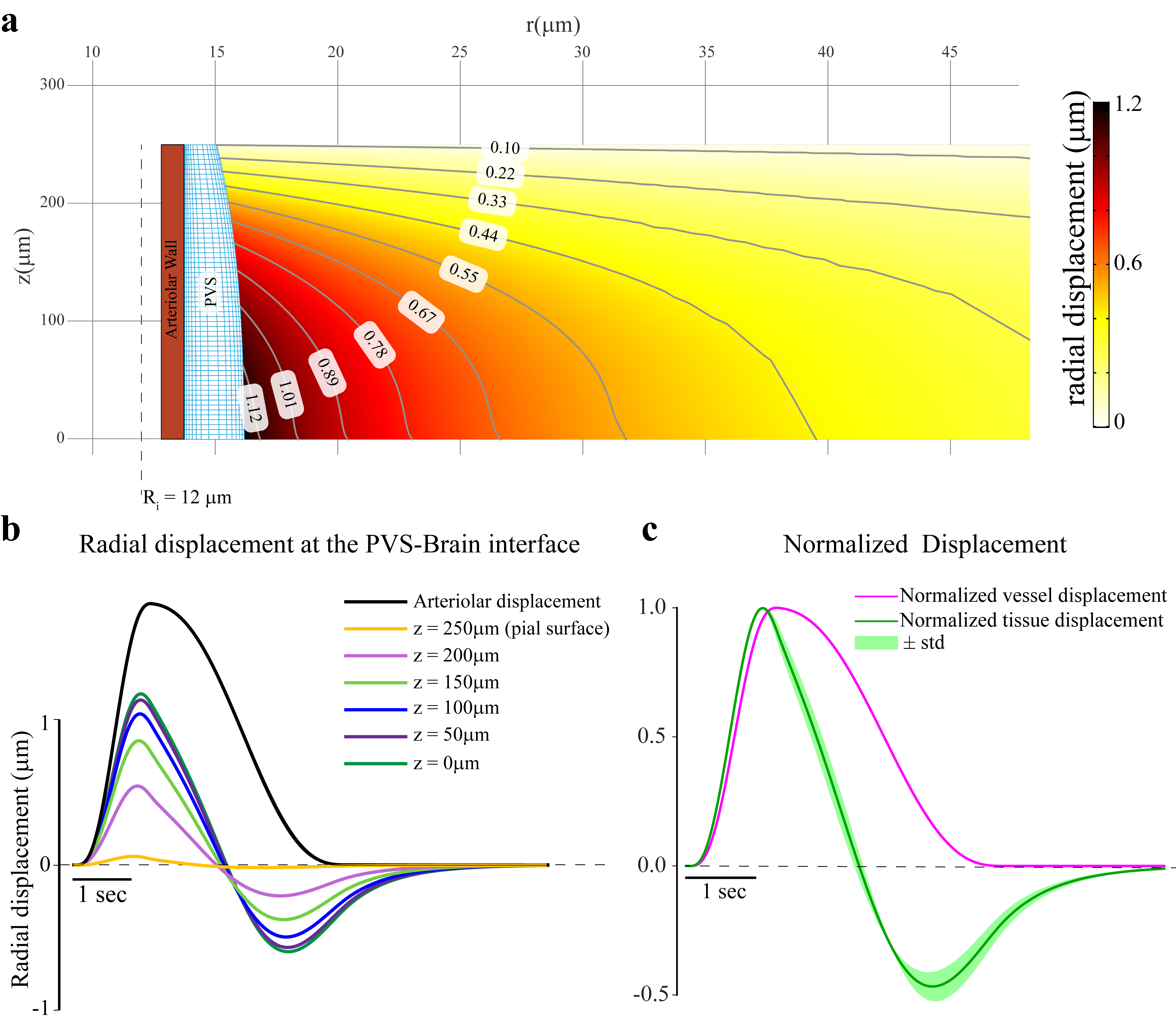

Supplement: Supplementary file 13 — Additional file 13: Figure S6. Deformation of the brain tissue due to the pressure changes in the PVS. Note the geometry is depicted with an unequal aspect ratio in the radial (r) and axial (z) directions for viewing convenience. a. Radial displacement contours in the brain tissue (maximum deformation, occurs at 1.16 seconds for the vasodilation profile shown in b). The brain tissue can deform by upto 1.2 µm, when the arteriole (with an initial radius of 12µm) increases its radius by 1.8 µm. b. Plot shows the change of radial displacement at the PVS-Brain interface with time. These deformations can be explained by the pressure changes in the PVS. When there is fluid outflow from the PVS, the increase in the pressure causes the brain tissue to deforms radially outward and when there is fluid influx, the brain tissue deforms radially inward. The smallest tissue displacement is at the pial surface (z = 250µm), which is the location of smallest pressure changes, as it is connected to the SAS flow resistance. The brain tissue is fixed at r = 150µm. c. Plot shows the change of radial displacement in the brain tissue at different distances from the centerline of the vessel. [file 12987_2020_214_MOESM13_ESM.tif]

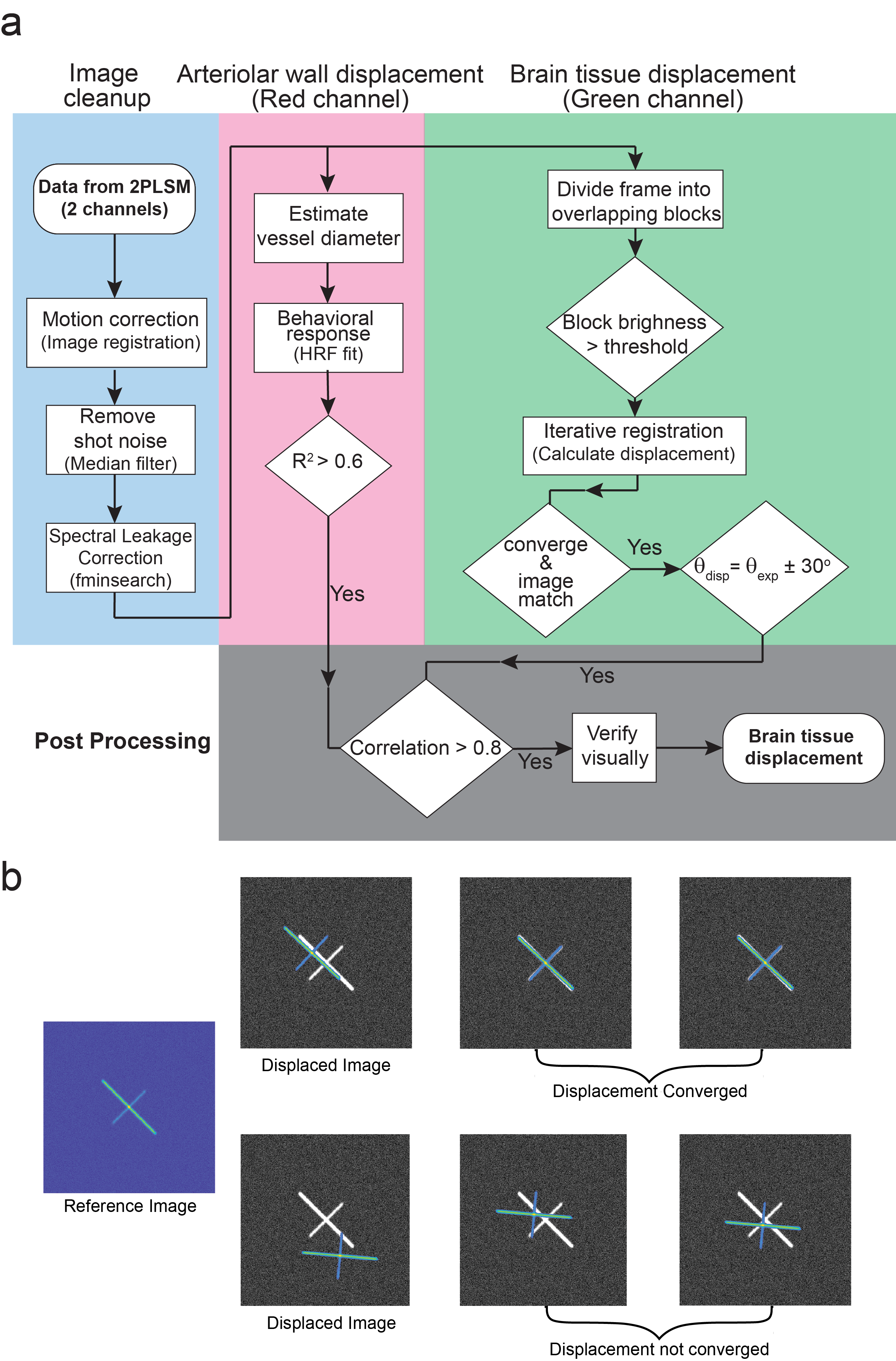

Supplement: Supplementary file 15 — Additional file 15: Figure S11. The procedure for measuring brain tissue displacement from in-vivo imaging data collected with a two-photon laser scanning microscope. a. Flow chart depicting the complete procedure used to calculate displacements in the brain tissue. The procedure can be broken down into 4 major sub-sections as shown in the figure. For a full description of the procedure, see methods. b. A depiction of the iterative method in calculating displacements. The figure on the left shows a reference image. The intensity is shown by a Parula colormap (Matlab). The images on the right show two cases of displaced images. The one on the top is rotated by 2°, and can be matched to the reference image (shown in gray) by a simple displacement. After the first calculation of the displacement and correcting the displaced image, the reference and the displaced image match and further iterations of displacement calculation yield a zero value, showing that the displacement calculation has converged. The one on the bottom is rotated by 45°, and cannot be matched to the reference image (shown in gray) by a simple displacement. In this case, every iteration of displacement calculation yields a non-zero value and the calculation is not converged. [file 12987_2020_214_MOESM15_ESM.tif]

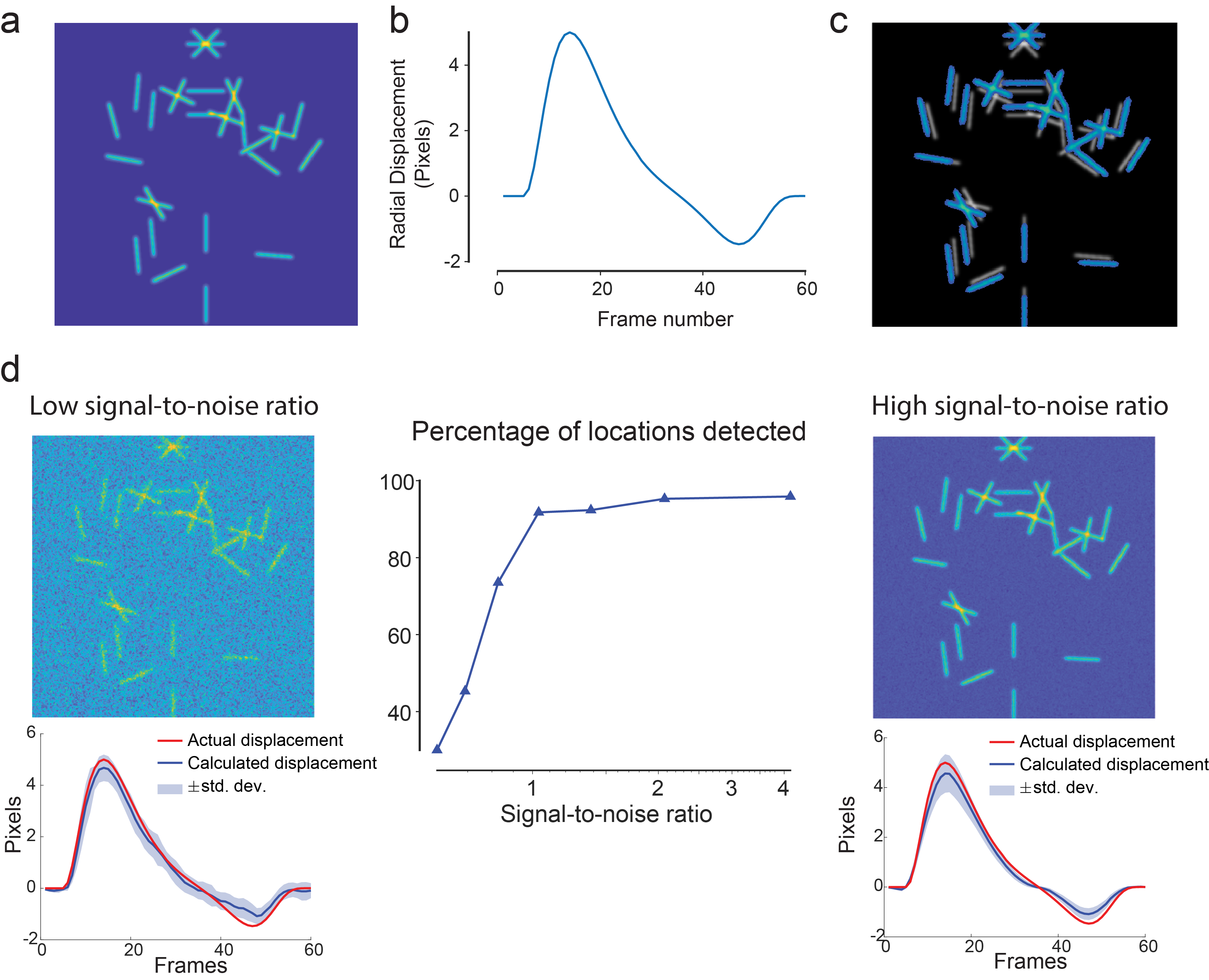

Supplement: Supplementary file 16 — Additional file 16: Figure S12. The displacement calculation method is robust to noise. a. A computer generated image (512 × 512 pixels) with randomly oriented lines. b. The radially-outward displacement given to the image shown in a. c. An image showing the radially-outward displacement at peak displacement (frame number 13). The initial position of the lines is shown in white and the displaced position is shown in blue. d. The displacement extraction procedure (shown in Figure S12) is robust to noise and predicts correct displacement. On the left, a case with low signal-to-noise ratio (0.59) is shown. The calculated displacements are very close to the actual displacement. The accuracy is comparable to the case with high signal-to-noise ratio (4.14) on the right. However, high noise results in a detection of displacement at fewer locations. The plot in the center shows that at low signal to noise ratio only 30% of the possible locations can be used for displacement calculations. Signal-to-noise ratio is calculated as the ratio of the mean signal value to the standard deviation in the noise. [file 12987_2020_214_MOESM16_ESM.tif]
